# The Complexity of Vesicle Transport Factors in Plants Examined by Orthology Search

**DOI:** 10.1371/journal.pone.0097745

**Published:** 2014-05-20

**Authors:** Puneet Paul, Stefan Simm, Oliver Mirus, Klaus-Dieter Scharf, Sotirios Fragkostefanakis, Enrico Schleiff

**Affiliations:** 1 Department of Biosciences Molecular Cell Biology of Plants; 2 Cluster of Excellence Frankfurt; 3 Center of Membrane Proteomics; Goethe University Frankfurt, Frankfurt/Main, Germany; Institut national de la santé et de la recherche médicale - Institut Cochin, France

## Abstract

Vesicle transport is a central process to ensure protein and lipid distribution in eukaryotic cells. The current knowledge on the molecular components and mechanisms of this process is majorly based on studies in *Saccharomyces cerevisiae* and *Arabidopsis thaliana*, which revealed 240 different proteinaceous factors either experimentally proven or predicted to be involved in vesicle transport. In here, we performed an orthologue search using two different algorithms to identify the components of the secretory pathway in yeast and 14 plant genomes by using the ‘core-set’ of 240 factors as bait. We identified 4021 orthologues and (co-)orthologues in the discussed plant species accounting for components of COP-II, COP-I, Clathrin Coated Vesicles, Retromers and ESCRTs, Rab GTPases, Tethering factors and SNAREs. In plants, we observed a significantly higher number of (co-)orthologues than yeast, while only 8 tethering factors from yeast seem to be absent in the analyzed plant genomes. To link the identified (co-)orthologues to vesicle transport, the domain architecture of the proteins from yeast, genetic model plant *A. thaliana* and agriculturally relevant crop *Solanum lycopersicum* has been inspected. For the orthologous groups containing (co-)orthologues from yeast, *A. thaliana* and *S. lycopersicum*, we observed the same domain architecture for 79% (416/527) of the (co-)orthologues, which documents a very high conservation of this process. Further, publically available tissue-specific expression profiles for a subset of (co-)orthologues found in *A. thaliana* and *S. lycopersicum* suggest that some (co-)orthologues are involved in tissue-specific functions. Inspection of localization of the (co-)orthologues based on available proteome data or localization predictions lead to the assignment of plastid- as well as mitochondrial localized (co-)orthologues of vesicle transport factors and the relevance of this is discussed.

## Introduction

**Figure 1:**
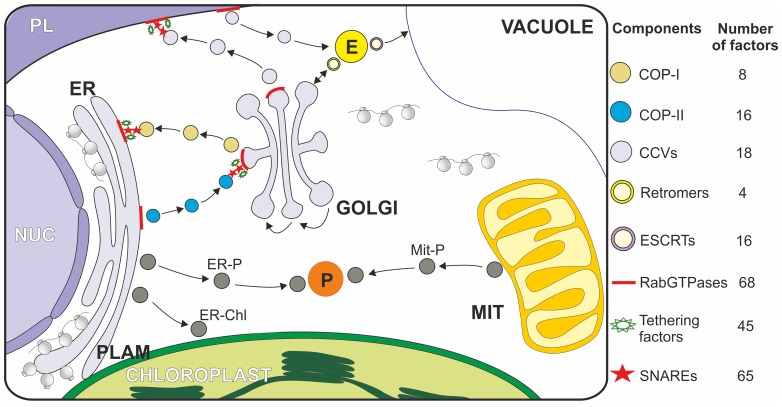
Vesicle transport pathways in plants. COP-II vesicles mediate cargo transport from ER to *cis-*Golgi, while COP-I traffics the cargo from Golgi to ER and intra-Golgi as well. Clathrin-coated vesicles (CCVs) are involved in flow of cargo from the plasma membrane and *trans*-Golgi network to endosomes and retromers and ESCRTs are required for endosomal trafficking pathways. Rab GTPases are involved in regulation of vesicle formation, its uncoating and transport, while tethering factors and SNAREs facilitate the membrane fusion processes. Additionally, vesicle transport has also been discussed between other compartments (shown in grey circles- not discussed in the manuscript). The number of identified factors for COP-II, COP-I, CCVs, Retromers and ESCRTs, Rab GTPases, Tethering factors and SNAREs is shown. MIT: mitochondria, ER: endoplasmic reticulum, E: endosome, P: peroxisome, NUC: nucleus, PLAM: Plastid Associated Membranes.

Vesicle transport ensures the exchange of macromolecules and proteins between different compartments and the endomembrane system. Membrane-bound vesicles mediate the transport of cargo from a donor to a target compartment [1]–[3]. Different routes have been identified (Fig. 1). The forward flow (anterograde) starts with vesicle transport from endoplasmic reticulum (ER) to Golgi, from which vesicles flow to various organelles and the plasma membrane (PM; secretory pathway). In addition, vesicles are transported from PM to vacuoles via endosomes (annotated as endocytic pathway) and a retrieval mechanism known as ‘retrograde pathway’, which delivers escaped proteins or lipids back to their residential compartments [4], [5]. Moreover, reports also suggest vesicle transport from ER to chloroplasts [6], ER to peroxisomes [7], [8] and mitochondria to peroxisomes [9]. However, ER - chloroplast (PLAM; Plastid Associated Membranes) and ER - mitochondria (MAMs; Mitochondrial Associated Membranes) contact sites are also discussed to function in lipid/protein and lipid exchange, respectively [10]–[12].

Each of the pathways involves a specific set of molecular processes acting in a series of events [13], [14]. The budding of the vesicle entails (i) selection of cargo followed by (ii) recruitment of the vesicle coat proteins and (iii) scission of the vesicle. The fusion of vesicle commences with (iv) its trafficking to target membrane along the cytoskeleton, (v) recognition of the vesicle at the target compartment by ‘tethering factors’ and (vi) the fusion of vesicle and the target membrane mediated by SNAREs ‘soluble NSF (*N*-ethylmaleimide sensitive factor) attachment protein receptors’. Besides the underlying commonality, distinctions exist in coat proteins and their recruitment processes, as well as in the involved regulatory GTPases, tethering factors, and the SNARE proteins.

Three major types of vesicles defined by their coat proteins are discussed: COP-II (coat protein complex-II), COP-I and Clathrin Coated Vesicles (CCVs; Fig. 1). COP-II vesicles mediate the flow from ER to *cis-*Golgi while COP-I vesicles account for the counter flow from Golgi to ER and intra-Golgi traffic [15]. CCVs are involved in the subsequent endocytic traffic flow [16]. In addition, retromer and ESCRT (endosomal sorting required for transport) coat complexes are also known to play a crucial role in endosomal trafficking pathways [17].

Similar to the animal and fungal system, plants have all the major components involved in vesicle-mediated transport [13], [18], [19]. It was noted that plants possess a high number of (co-)orthologues for the respective factors; coat proteins, Rab GTPases, SNAREs, etc. [13]. It is also discussed that the plant secretory system possesses certain distinctive features in comparison to the yeast, namely the absence of the ER-Golgi intermediate compartment (ERGIC), a drastically reduced mobility of Golgi stacks [20], and an activity of the *trans*-Golgi network (TGN) as an early endosome [13] to name a few.

At present, majority of the knowledge concerning vesicle transport in plants has been conducted for the model plant *A. thaliana* [13], [14], [18], [19], [21]. Thus, we used the available information on vesicle transport factors from the model systems *A. thaliana* and yeast to define orthologous groups from the proteomes of 14 different plant species. We discuss the results with a special focus on agriculturally relevant crop plant *Solanum lycopersicum* (tomato), which represents the model plant system for studying fleshy fruit development, ripening and wound response [22]–[24]. However, we did not inspect the time point of duplication in relation to speciation, because definition of paralogues [25] was not in focus of our analysis. Thus, we used the term orthologue for representing genes of two different species derived from a single common ancestor, while the term (co-)orthologue has been used to designate the orthologous relationships due to lineage-specific duplication [26]. The detection of (co-)orthologues was achieved by the bi-directional BLAST-dependent orthologue search algorithms OrthoMCL and PGAP. The experimentally proven and bioinformatically predicted vesicular transport proteins of *A. thaliana* and yeast corresponding to ‘core-set’ of 240 factors were used as bait to detect putative proteins and group of (co-)orthologues. The (co-)orthologues were discussed in some detail for the model systems yeast, *A. thaliana* and *S. lycopersicum* concerning domain architecture and intracellular localization, while the tissue-specific expression analysis was performed for the two plant species. Based on our results, we provide an overview concerning conservation and diversification of orthologues to factors involved in the vesicle transport systems in Viridiplantae.

## Materials and Methods

### Database composition and orthologue search

Literature search for proteins involved in vesicle transport was performed for the two model systems *S. cerevisiae* and *A. thaliana* as described [27]. Manual confirmation of the yeast and *A. thaliana* proteins described to be involved in the vesicular transport was performed by screening existing literature for each single protein based on the SGD (http://www.yeastgenome.org/; Table S1 [28]–[109]) and TAIR (http://www.arabidopsis.org/; Table S2 [21], [110]–[206]) databases. The protein sequences were categorized as bioinformatically predicted or experimentally proven. For all identified factors in *S. cerevisiae* and *A. thaliana* the corresponding protein sequences were extracted from http://www.yeastgenome.org (*S. cerevisiae -* April 2012) and http://www.arabidopsis.org (*A. thaliana -* TAIR10).

Orthologue identification is based on the strategy defined by Paul et al. [27], which used two different orthologue search algorithms for 14 plant genomes and yeast. These different algorithms are based on different approaches. The combination of OrthoMCL and PGAP were used in order to improve the accuracy of detecting false positives and false negatives. In brief, the PGAP (**p**an **g**enome **a**nalysis **p**ipeline) in which InParanoid and MultiParanoid (—method MP) are implemented was used to cluster sequences of *S. cerevisiae, A. thaliana* and *S. lycopersicum* (ITAG2.3 http://solgenomics.net) in their respective orthologous groups [207].

Orthologue identification in *S. cerevisiae, A. thaliana, S. lycopersicum* and 12 other plant species was performed using OrthoMCL [208] to identify orthologous groups for more than three species in a less time-consuming clustering and also to compare the different predictions. The plant genomes were extracted from (i) *B. distachyon* (bradi1.2 with GAEVAL http://www.plantgdb.org), (ii) *C. reinhardtii* (JGI v4 with GAEVAL http://www.plantgdb.org), (iii) *G. max* (Glyma1 http://www.plantgdb.org), (iv) *L. japonicus* (Lj1.0 http://www.plantgdb.org), (v) *M. truncatula* (Mt3.5v5 http://jcvi.org), (vi) *O. sativa* (MSU Version 7.0 with GAEVAL http://www.plantgdb.org), (vii) *P. patens* (Phypa1.6 http://phytozome.net), (viii) *P. trichocarpa* (Ptr v2.0 with GAEVAL http://www.plantgdb.org), (ix) *S. tuberosum* (PGSC v3.4 http://potatogenome.net), (x) *S. bicolor* (JGI Sbi1 http://www.plantgdb.org), (xi) *V. vinifera* (Genescope 12X http://genoscope.cns.fr), and (xii) *Z. mays* (B73 RefGen v2 http://www.plantgdb.org). All genomes downloaded from PlantGDB [209] have verified annotations of genes in relation to alternative splicing and gene fusions/fissions by ‘gene annotation evaluation algorithm’ (GAEVAL) [210]. OrthoMCL filtered away nine poor-quality sequences by our evaluation process based on the protein sequence length (<10 amino acids) and percent of stop codons (marked by asterisks; >20%). The results derived from both orthologue prediction algorithms (OrthoMCL, PGAP) were used to check for consistency and automatically combined to generate the list of the vesicle transport components in yeast, *A. thaliana* and *S. lycopersicum*. For all other plant species we only rely on the results of OrthoMCL.

### Domain analysis

Protein family scan from Pfam (Version 26.0) [211] was performed to predict functional domains of the protein sequences comprising different vesicle components. Moreover, order and similarity of domains of the (co-)orthologues in a respective orthologous group was analyzed automatically by customized Python scripts (www.python.org). The name of the Pfam domain is indicated when discussed and the description of the individual domains is available in the Pfam database (http://pfam.sanger.ac.uk/). The comparison of domains of (co-)orthologues within one orthologous group was done in relation to the detected domains and their order of occurrence. Based on this, we distinguished three classes; the first class (Class I) means the similar domains and their identical order of occurrence. Class II means that additional parts or at least some of the domains occur in both orthologues referring to their partial similarity in domain architecture, whereas class III means that both orthologues share no similarity in their domain architecture. For comparison of domains in the respective orthologous groups, we used bait as starting point for our analysis, which is classified concerning their reliability to be involved in vesicular transport based on experimentally proven or bioinformatically predicted proteins of yeast and *A. thaliana*. The major bait of each orthologous group is marked with an asterisk (*) and the minor baits are marked with plus (+).

### Localization prediction

Localization analysis for (co-)orthologues of the identified factors was performed with a high certainty approach for *A. thaliana*, while a low certainty procedure was undertaken for other plant species and yeast, because for the latter only predictors that allowed massive sequence analysis were used.

#### High certainty approach

The prediction was based on publically available experimental data; Green Fluorescent Protein (GFP) based localization studies and mass spectrometry (MS) data. Further, experimental information for chloroplast and mitochondria localized (co-)orthologues (Table S3, Table S4) as well as for the other compartments was extracted from SUBA3 [212], FTFLP [213] and PPDB [214]. This information was used to build a consensus on the majority basis. All (co-)orthologues without experimentally confirmed localization were assigned to a particular compartment using 20 different localization predictors provided by SUBA3 [212], which represents the consensual localization via bare majority. Additionally, we utilized the annotation provided by TAIR as well as in the literature based on experimental studies for individual protein with respect to their localization to verify the localization data of the high throughput analyses via mass spectrometry or GFP fluorescence.

#### Low certainty approach

For other plant species, experimental evidences for intracellular localization are largely absent. Thus, we selected YLoc, WoLF PSORT, TargetP, Predotar, MitoPred and ChloroP from SUBA3 localization predictor bundle, which enable the automation of the localization approach by submitting ≥2 sequences at once. The predictor YLoc [215] and WoLF PSORT [216] distinguish between 11 different compartments (extracellular, nucleus, Golgi, ER, mitochondrion, plastid, plasma membrane, peroxisome, vacuole, cytosol and cytoskeleton), while TargetP [217] and Predotar [218] are highly accepted to distinguish between chloroplast, mitochondria and secretory pathway localization. In addition to the multi-compartment localization predictor, we use MitoPred [219] as mitochondrial specific and ChloroP [220] as chloroplast specific localization predictor to strengthen the results, because both predictors are specifically trained to detect proteins with the respective signals. The localization results of YLoc and WoLF PSORT for vacuole, ER, Golgi, plasma membrane are merged and represented as endomembranes.

### Cluster analysis of expression data

We downloaded microarray expression data from nine different tissues for *A. thaliana* (Table S5); (i) flower (4 samples, GSE32193); (ii) fruit (3 samples, GSE28446); (iii) ovules (2 samples, GSE27281); (iv) mature pollen (4 samples, GSE17343); (v) root (2 samples, GSE21504); (vi) anther (3 samples, GSE18225); (vii) seedlings and whole plant (23 samples, GSE5629); (viii) shoot and stem (41 samples, GSE5633); and (ix) leaf (59 samples, GSE5630) while for *S. lycopersicum* seven different tissues (Table S5) were considered (GSE19326, (i) cotyledons: 2 samples; (ii) hypocotyledons: 2 samples; (iii) 3-weeks old leaves: 3 samples; (iv) 5-weeks old leaves: 3 samples; (v) roots: 3 samples and GSE22300, (vi) fruit: 3 samples; (vii) flower: 1 sample). The raw CEL data of the samples of both organisms were normalized using the APT (Affymetrix Power Tools) software package [221] with RMA (Robust Multichip Average) [222]. Further, to avoid overweighting of certain tissues with multiple samples we considered mean expression level from a maximal number of four samples for each tissue by performing hierarchical clustering. The RMA normalized expression data from a maximum of four samples per tissue of both organisms were used to build the average, which was used to cluster independently by using a k-means clustering algorithm (Pycluster 1.50). The number of clusters (k) for the k-means clustering was limited to 10, which was determined by performing the clustering for 1 to 50 clusters and then plotting the distance to the optimal solution (Fig. S1) (iii) the available Affymetrix IDs for the vesicle transport proteins from the GeneChips of *A. thaliana* (GPL198 alias ATH1-121501) and *S. lycopersicum* (GPL4741) were identified and used for clustering the genes encoding vesicle transport proteins (iv) for detecting the expression for different tissues more easily the median of the samples concerning the tissues and clusters was determined.

## Results

### Bioinformatic detection of orthologues to factors involved in vesicle transport

We performed literature search for factors involved in vesicle transport pathways and extracted 212 factors corresponding to different pathways in *A. thaliana* [14], [21], [147], [177] and 45 factors in *S. cerevisiae* [223], [224]. From the initial set of 257 factors, we realized an overlap of 17 factors identified for both species thus yielding 240 different factors used as ‘core-set’. The ‘core-set’ contains 8 factors for the COP-II, 16 for COP-I, 18 for Clathrin Coated Vesicles (CCV), 20 for Retromers and ESCRTs, 68 for Rab GTPases, 45 for Tethering factors and 65 for SNAREs (Fig. 1, Table 1–7, Table S6–S19). The ‘core-set’ was further analyzed to discriminate between experimentally proven or bioinformatically predicted protein sequences (Table S1 [28]–[109], Table S2 [21], [110]–[206]). The same holds true for the (co-)orthologues identified for yeast and *A. thaliana* described below, for which existing literature was screened using SGD (http://www.yeastgenome.org/; Table S1 [28]–[109]) and TAIR (http://www.arabidopsis.org/; Table S2 [21], [110]–[206]).

**Table 1:** The number of identified orthologues to COP-II-coated vesicle components.

Complex	Factor	Bait			yeast		Arabidopsis		tomato	
		yeast	Ara (Exp.)	Ara (Pred.)	I	II	I	II	I	II
Coatomer Sec13/31 (cage)	Sec13	*	+		1	1	1	3	1	3
				*			1		1	
	Sec31	*	+		1			2		1
		*	+		1		1		1	
			*				3		2	1
Coatomer Sec23/24 (cargo selective)	Sec23	*		+	1		5		4	
				*			1			1
	Sec24	*	+		2		1			2
			*	+			2		2	
				*			1			1
GEF	Sec12 & Sed14	*	+		2			3		2
GTPase	Sar1-like	*	+	+	1		4		4	
			*				1		1	
				*			1			
	Sec16		*				1	1	2	
		*			1					
TOTAL	8				10	1	23	9	18	11

The yeast and Arabidopsis proteins are used as bait to assign classification based on domain architecture (classes I and II, III is not populated and thus omitted; see Material and Methods). Given are the complex and factor (column 1 and 2) as well as the major bait (*) and minor bait (+) in the orthologous groups of a respective factor. The major bait (*) was chosen from yeast or Arabidopsis proteins due to their reliability to be involved in vesicular transport; and the order for choosing the bait is yeast proteins > Arabidopsis experimentally proven proteins (exp.) > Arabidopsis predicted proteins (pred.). Accession numbers and amino acid length of the proteins are listed in Table S13 for the respective species.

**Table 2:** The number of identified orthologues to COP-I-coated vesicle components.

Complex	Factor	Bait			yeast	Arabidopsis		tomato	
		yeast	Ara (Exp.)	Ara (Pred.)	I	I	II	I	II
B-COP (cage)	α (RET1p)	*		+	1	2		2	
	β′ (Sec27p)	*		+	1	2	1	2	1
	ε (Sec28p)	*		+	1	2		1	
F-COP (cargo selective)	β (Sec26p)	*		+	1		2		2
	Υ (Sec21p)	*	+		1	1		1	
	δ (RET2p)	*		+	1		1		2
	ζ (RET3p)	*	+	+	1	3		3	
GEF	Sec7-type	*	+		1	5		3	
	GNOM-type	*	+		1		3		5
GTPaseARF	ARF1A	*	+	+	2	6		4	
	ARF1B			*		2		2	
				*		1			
	ARF1C			*		1		1	
	ARF1D			*		2			
GTPases ARF-like	ARLA			*		3		4	
				*		1			
	ARLB	*		+	1	1		1	
	ARLC			*		1		1	
TOTAL	16				12	33	7	25	10

The yeast and Arabidopsis proteins are used as bait to assign classification based on domain architecture (classes I to III; not populated classes are not shown; see Material and Methods). Given are the complex and factor (column 1 and 2) as well as the major bait (*) and minor bait (+) in the orthologous groups of a respective factor. The major bait (*) was chosen from yeast or Arabidopsis proteins due to their reliability to be involved in vesicular transport; and the order for choosing the bait is yeast proteins > Arabidopsis experimentally proven proteins (exp.) > Arabidopsis predicted proteins (pred.). Accession numbers and amino acid length of the proteins are listed in Table S14 for the respective species.

**Table 3:** The number of orthologues for Clathrin-Coated Vesicle (CCVs) transport factors.

Complex	Factor	Bait			yeast	Arabidopsis		tomato	
		yeast	Arabi (Exp.)	Arabi (Pred.)	I	I	II	I	II
Triskelion (cage)	Heavy chain	*	+		1		2		3
	Light chain		*			2		1	
		*			1				
AP1	γ	*	+		1	1	1	2	
	β1 & β 2′	*		+	1		2		1
	µ1	*	+		1	2		2	
	σ1	*	+	+	1	2		1	
AP2	α	*	+		1		2		3
	µ2		*			1		2	
	σ2	*		+	1	1		1	
AP3	δ	*	+		1	1		1	
	β3	*	+		1	1		1	
	µ3			*		1		1	
	σ3	*		+	1	1		2	
AP4	ε			*		1			
	β4			*		1		1	
	µ4 & σ4		*			1		1	
TOTAL	18				11	16	7	16	7

The yeast and Arabidopsis proteins are used as bait to assign classification based on domain architecture (classes I to III; see Material and Methods). Given are the complex and factor (column 1 and 2) as well as the major bait (*) and minor bait (+) in the orthologous groups of a respective factor. The major bait (*) was chosen from yeast or Arabidopsis proteins due to their reliability to be involved in vesicular transport; and the order for choosing the bait is yeast proteins > Arabidopsis experimentally proven proteins (exp.) > Arabidopsis predicted proteins (pred.). Accession numbers and amino acid length of the proteins are listed in Table S15 for the respective species.

**Table 4:** Number of orthologues to Retromer and ESCRT transport factors.

Complex	Factor	Bait			yeast		Arabidopsis		tomato	
		yeast	Arabi (Exp.)	Arabi (Pred.)	I	II	I	II	I	II
PIP3P-binding	Vps5/SNX	*	+		2	1	3		2	1
Cargo recognition	VPS26	*	+		1		2		1	
	VPS29	*	+		1		1		1	
	VPS35	*	+		1		3		3	
ESCRT-I	VPS23	*	+	+	1		2	1	3	
	VPS28	*	+	+	1		2		2	
	VPS37			*			2		2	
ESCRT-II	VPS22	*	+		1		1		1	
	VPS25	*		+	1		1		1	
	VPS36			*			1		1	
AP4	VPS2	*	+		1		1		1	
	VPS20 & SNF7a	*	+	+	1		2		1	
	VPS24	*		+	1		2		2	
	SNF7b & SNF7c	*	+		1		2		3	
	DID2	*	+		1		2		2	
Misc.	VPS31/Bro1	*	+		1		1		2	
	VPS4	*	+		1		1		3	
	Hrs/VPS27			*			1		1	
TOTAL	20				16	1	30	1	32	1

The yeast and Arabidopsis proteins are used as bait to assign classification based on domain architecture (classes I to III; see Material and Methods). Given are the complex and factor (column 1 and 2) as well as the major bait (*) and minor bait (+) in the orthologous groups of a respective factor. The major bait (*) was chosen from yeast or Arabidopsis proteins due to their reliability to be involved in vesicular transport; and the order for choosing the bait is yeast proteins > Arabidopsis experimentally proven proteins (exp.) > Arabidopsis predicted proteins (pred.). Accession numbers and amino acid length of the proteins are listed in Table S16 for the respective species.

**Table 5:** The number of Rab GTPase orthologoues.

Complex	Factor	Bait			yeast	Arabidopsis	tomato
		yeast	Arabi (Exp.)	Arabi (Pred.)	I	I	I
Rab group	RABA1a-e, g-i; RABA2a-b	*	+	+	2	10	11
	RABA1f			*		1	2
	RABA2c-d			*		2	1
	RABA3			*		1	1
	RABA4a			*		1	2
	RABA4b			*		1	
	RABA4c-d		*	+		2	3
	RABA4e			*		1	
	RABA5a			*		1	2
	RABA5b			*		1	1
	RABA5c-e		*	+		3	1
	RABA6a-b			*		2	1
	RABB1a			*		1	
	RABB1b		*			1	2
	RABB1c			*		1	1
	RABC1			*		1	1
	RABC2a		*			1	2
	RABC2b			*		1	
	RABD1		*			1	1
	RABD2a-c	*	+		1	3	4
	RABE1a, c-e	*	+	+	1	5	5
	RABE1b		*			1	2
	RABF1, RABF2a-b	*	+		3	3	4
	RABG1			*		1	
	RABG2, RABG3a-f	*	+	+	1	7	4
	RABH1a-e	*	+	+	1	5	3
Other small GTPases	Arf1-l2	*	+		1	1	1
	ArfRP1-l	*		+	1	1	1
	Arl2-like			*		1	1
	Arl5-like			*		1	1
	Arl8-like			*		3	4
				*		1	
PM (SM family)	KEULLE			*		1	
	SEC1a-b	*	+	+	1	3	2
TGN(SM)	VPS45	*	+		1	1	1
LE/vac. (SM)	VPS33	*		*	1	1	1
ER-Golgi (SM)	SLY1	*		*	1	1	1
TOTAL	68				15	73	67

The yeast and Arabidopsis proteins are used as bait to assign classification based on domain architecture (classes I to III; see Material and Methods). Given are the complex and factor (column 1 and 2) as well as the major bait (*) and minor bait (+) in the orthologous groups of a respective factor. The major bait (*) was chosen from yeast or Arabidopsis proteins due to their reliability to be involved in vesicular transport; and the order for choosing the bait is yeast proteins > Arabidopsis experimentally proven proteins (exp.) > Arabidopsis predicted proteins (pred.). Accession numbers and amino acid length of the proteins are listed in Table S17 for the respective species.

**Table 6:** The orthologues of Tethering factors.

Complex	Factor	Bait			yeast			Arabidopsis			tomato		
		yeast	Arabi (Exp.)	Arabi (Pred.)	I	II	III	I	II	III	I	II	III
Coiled coils	Uso1	*	+		1			1			1		
	COY1	*	+		1			1			1		
	Rud3/Grp1	*			1								
	Imh1	*			1								
HOPS	VPS11	*	+		1				1			1	
	VPS16	*	+		1			1			1		
	VPS18	*		+	1				1			1	
	VPS33	*	+		1			1			1		
	VPS39	*		+	1				1			2	
	VPS41	*		+	1			1				2	
CORVET	VPS8	*	+		1				1			1	
	VPS3	*			1								
CATCHR family Exocyst	SEC3	*			1								
			*	+				2			1		
	SEC5	*			1			2			2		
	SEC6	*			1			1			1		
	SEC8	*			1								
			*					1			1		
	SEC10	*			1				1			4	
	SEC15	*			1			2			2		
	EXO70	*	+		1			14			12		
	EXO84	*			1								
			*					1			1		
CATCHR/DSL1 complex (MTC)	Tip20	*			1								
			*					1					
	DSL1	*			1								
	SEC39	*			1								
CATCHR family COG complex	COG1	*			1								
				*				1			1		
	COG2	*			1								
				*				1			1		
	COG3	*		+	1			1			1		
	COG4	*		+	1				1			1	
	COG5	*			1								
				*				1			1		
	COG6	*		+	1			1			1		
	COG7	*			1								
				*				1			1		
	COG8	*			1								
				*				1			1		
CATCHR family GARP complex	VPS52	*			1			2			1		
	VPS53	*			1			2			1		
	VPS54	*			1				1				2
	VPS51	*			1								
TRAPP-I	Bet3	*		+	1			1			2		
	Bet5	*		+	1			1			1		
	Trs20	*		+	1			1			1		
	Trs23	*		+	1				1			1	
	Trs31	*		+	1			1			1		
	Trs33	*		+	1			1			1		
	Trs85	*			1								
TRAPP-II	Trs120	*			1								
			*					1			1		
	Trs130	*			1								
				*				1			1		
	Trs65	*			1								
TOTAL	45				45			47	8		41	13	2

The yeast and Arabidopsis proteins are used as bait to assign classification based on domain architecture (classes I to III; see Material and Methods). Given are the complex and factor (column 1 and 2) as well as the major bait (*) and minor bait (+) in the orthologous groups of a respective factor. The major bait (*) was chosen from yeast or Arabidopsis proteins due to their reliability to be involved in vesicular transport; and the order for choosing the bait is yeast proteins > Arabidopsis experimentally proven proteins (exp.) > Arabidopsis predicted proteins (pred.). Accession numbers and amino acid length of the proteins are listed in Table S18 for the respective species.

**Table 7:** The number of SNARE orthologues detected.

Complex	Factor	Bait			yeast			Arabidopsis			tomato		
		yeast	Arabi (Exp.)	Arabi (Pred.)	I	II	III	I	II	III	I	II	III
ER(Qa)	SYP81			*				1			2		
Golgi(Qa)	SYP31 & SYP32	*	+		1				2			2	
TGN(Qa)	SYP41–43	*	+		1				3			2	
Late Endosome/vac. (Qa)	SYP21–23	*	+		1				3		1	4	
	SYP24		*					1					
Cell plate (Qa)	KNOLLE!	*	+	+	1	1		7			6		
	SYP112			*				1			1		
PM1(Qa)	PEN1		*					1			2		
	SYP122!	Together in group of KNOLLE											
PM2(Qa)	SYP123!	Together in group of KNOLLE											
	SYP124 & SYP125	Together in group of KNOLLE											
PM3(Oa)	SYP131 & SYP132	Together in group of KNOLLE											
ER(Qb)	SEC20		*					1			1		
ER/Golgi(Qb)	MEMB11& MEMB12		*					2			1		
Golgi(Qb)	GOS11		*					1			1		
	GOS12	*	+		1			1			1		
TGN/Vac. (Qb)	VTI11		*					1	1		1		
	VTI12		*	+				7	11			4	
	VTI13 & VTI14	*	+		1			3			1		
TGN/PM(Qb)	NPSN11		*					1			1		
	NSPN12 & NSPN13		*	+				2					1
ER(Qc)	USE11 & USE12			*				2			1		
ER/Golgi(Qc)	BET11 & BET12		*	+				2			2		
Golgi(Qc)	SFT11 & SFT12	*	+	+	1					2			1
TGN/Vac. (Qc)	SYP51 & SYP52	*	+	+	1				2			2	
TGN/Endo (Qc)	SYP61	*	+		1			1					3
PN(Qc)	SYP71 & SYP72			*				1		1	1		
	SYP73		*					1					
PM(Qb+c)	SNAP33			*				1				2	
	SNAP29			*				1					
	SNAP30			*				1					
ER/Gol.(R)	SEC22	*	+		1			1			3		
Golgi/Vac(R)	YKT61, 62	*	+		1			2			1		
TGN/Vac(R) & TGN/PM1(R)	VAMP711–714 & VAMP723 & VAMP728	*	+	+	2			1	5			5	
	VAMP722 & VAMP725 & VAMP726		*	+				3	1		3		
	VAMP721		*					1					
TGN/PM2 (R)	VAMP724			*				1			1		
TGN/PM3 (R)	VAMP727		*					1			1		
PM (R)	TYN11 & TYN12	*		+	2					2		1	3
Disassembly unit	SEC18	*	+		1			1				1	
	SEC17	*		+	1					2			2
TOTAL	65!				17	1		51	28	7	32	23	10

The yeast and Arabidopsis proteins are used as bait to assign classification based on domain architecture (classes I to III; see Material and Methods). Given are the complex and factor (column 1 and 2) as well as the major bait (*) and minor bait (+) in the orthologous groups of a respective factor. The major bait (*) was chosen from yeast or Arabidopsis proteins due to their reliability to be involved in vesicular transport; and the order for choosing the bait is yeast proteins > Arabidopsis experimentally proven proteins (exp.) > Arabidopsis predicted proteins (pred.). Accession numbers and amino acid length of the proteins are listed in Table S19 for the respective species.

**Figure 2:**
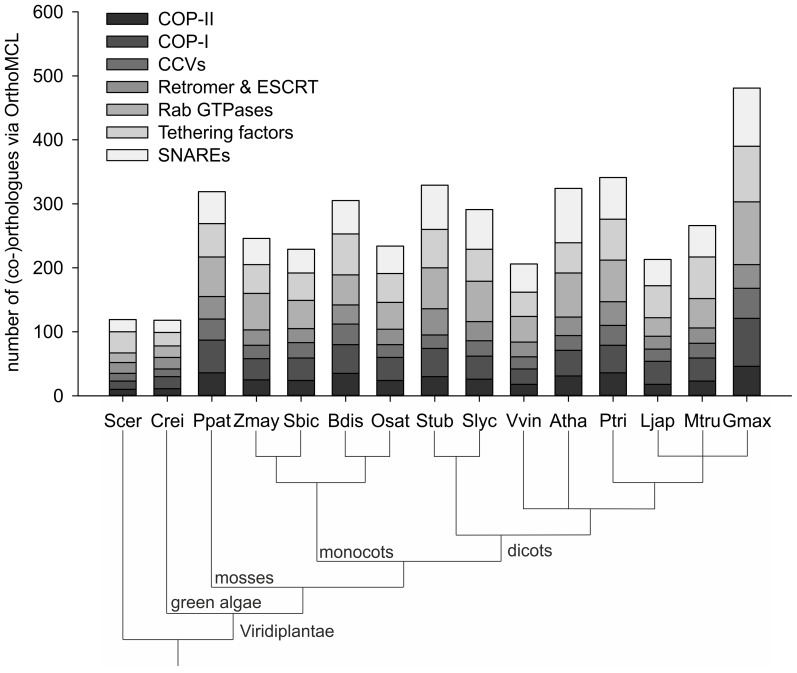
Correlation of protein sequences and orthologue number. Shown are the number of orthologues for different vesicle transport subfamilies of yeast, *A. thaliana* and 13 other plant species in accordance to their phylogenetic relationship. (Scer: *S. cerevisae*; Crei: *C. reinhardtii*; Ppat: *P. patens*; Zmay: *Z. mays*; Sbic: *S. bicolor*; Bdis: *B. distachyon*; Osat: *O. sativa*; Stub: *S. tuberosum*; Slyc: *S. lycopersicum*; Vvin: *V. vinifera*; Atha: *A. thaliana*; Ptri: *P. trichocarpa*; Ljap: *L. japonicus*; Mtru: *M. truncatula*; Gmax: *G. max*).

The ‘core-set’ of factors was used to detect the likely orthologous groups in the 14 analyzed plant genomes and *S. cerevisiae* via ‘OrthoMCL’ (Fig. 2, Table S6–S12). The proteome sequences of the species are subjected to an all-against-all BLASTP to find reciprocal best similarity pairs between species (putative orthologues) and reciprocal best similarity pairs within species (putative co-orthologues). Both pairs are used to define species normalized similarity matrices, which are then used to classify orthologous groups via Markov clustering. Consequently, we identified 4021 different (co-)orthologues corresponding to 14 plant genomes via OrthoMCL search. For most of the plant genomes the number of (co-)orthologues ranges between 200 and 300, whereas yeast and *Chlamydomonas reinhardtii* contains nearly 120 (co-)orthologues and *Glycine max* nearly 500 (Fig. 2).

In general, 150 of the initial set of 240 vesicle transport factors are conserved in algae (*C. reinhardtii*), moss (*P. patens*), monocots and dicots, whereas eight tethering factors (RUD3, IMH1, VPS3, DSL1, SEC39, VPS51, TRS85, TRS65) could only be identified in yeast. For the majority of the analyzed factors at least one (co-)orthologue is observed in most of the analyzed plants. Moreover, multiple (co-)orthologues have been found in the analyzed plant species for most of the vesicle transport factors (Table S6–S12). In turn, orthologues to 29 factors are only absent in *C. reinhardtii,* while orthologues to 15 factors seem to be only present in monocots and dicots. Interestingly, orthologues to 31 factors seem to be specific for *A. thaliana* or dicots in general (Table S19).

In addition, ‘PGAP’ with implemented InParanoid and MultiParanoid-like algorithms (see Materials and Methods) was employed to complement the OrthoMCL analysis in case of *S. cerevisiae*, *A. thaliana* and *S. lycopersicum* (Table S13–S19). We combine the results of both algorithms to reduce the number of false negatives. The algorithm uses the pairwise similarity scores between two species based on an all-against-all BLASTP. The constructed orthologous groups consist of two seed orthologues identified by a reciprocal best-hit search between two organisms. Further, more sequences are added to the orthologous group on basis of their similarity to the corresponding seed orthologue. The pairwise orthologous groups of more than two species are merged concerning their overlap.

Both BLAST-dependent orthologue search algorithms perform an all-versus-all BLAST of the protein sequences to detect pairs, which is more sensitive and reliable than a unidirectional BLAST search. Further, the orthologue search was used to detect groups of orthologous genes from different plant species, which allowed the detection of so called (co-)orthologues due to lineage-specific duplications [26]. Consequently, we identified 129 different (co-)orthologues for *S. cerevisiae* corresponding to 171 factors of the ‘core-set’ of 240 factors, because some of the (co-)orthologues of different vesicular transport factors fall in the same orthologous groups. The 340 and 307 different (co-)orthologues for *A. thaliana* and *S. lycopersicum* could be assigned to 231 and 223 factors, respectively. The genes not related to the vesicle transport are discussed in the following sections.

### Domain analyses of identified orthologues to vesicle factors

Orthologues typically perform equivalent functions (Koonin, 2005), but they are not necessarily involved in the same cellular process. However, if in addition to the inferred orthology the same domain architecture and same protein localization is observed, the likelihood that the identified protein performs the function in a similar cellular process as the bait is very high (e.g. [225]). Thus, we inspected the domain architecture of the proteins from *S. cerevisiae, A. thaliana* and *S. lycopersicum* as an additional hint for an involvement of the identified (co-)orthologues in vesicle transport (Table S21–S27).

For the analysis of the domain architecture, we used bait on the basis of its reliability for being involved in vesicular transport as per existing literature (Table S1 [28]–[109], Table S2 [21], [110]–[206]). Thus, (co-)orthologues with an experimental proven evidence is preferentially used as major bait, while bioinformatically predicted protein sequences are only used as major bait (*) in the case where no experimental evidence is available for the orthologue in the respective group (Tables 1–7). In case, when >1 bait have been identified, we used the sequence of the yeast proteins as major bait (*) and the (co-)orthologues of *A. thaliana* as minor bait (+).

Further, for analyzes of the domain architecture of the orthologues in different orthologous groups, three classes have been defined (see Materials and Methods). Starting from the major bait (*) the domain architecture of all other (co-)orthologues within one group were compared to the major bait and classified accordingly. For the orthologous groups containing (co-)orthologues from yeast, *A. thaliana* and tomato, we observed the same domain architecture (class I) for ∼79% (416/527) of the (co-)orthologues, which indicates a very high conservation of this process (Tables 1–7). Overall, analyzes of domain architecture of detected 776 (co-)orthologues in the three species (*A. thaliana, S. lycopersicum, S. cerevisiae*) lead to the assignment of 629 (co-)orthologues to class I and 127 (co-)orthologues to class II using the respective major bait for the domain annotation (Tables 1–7).

**Figure 3:**
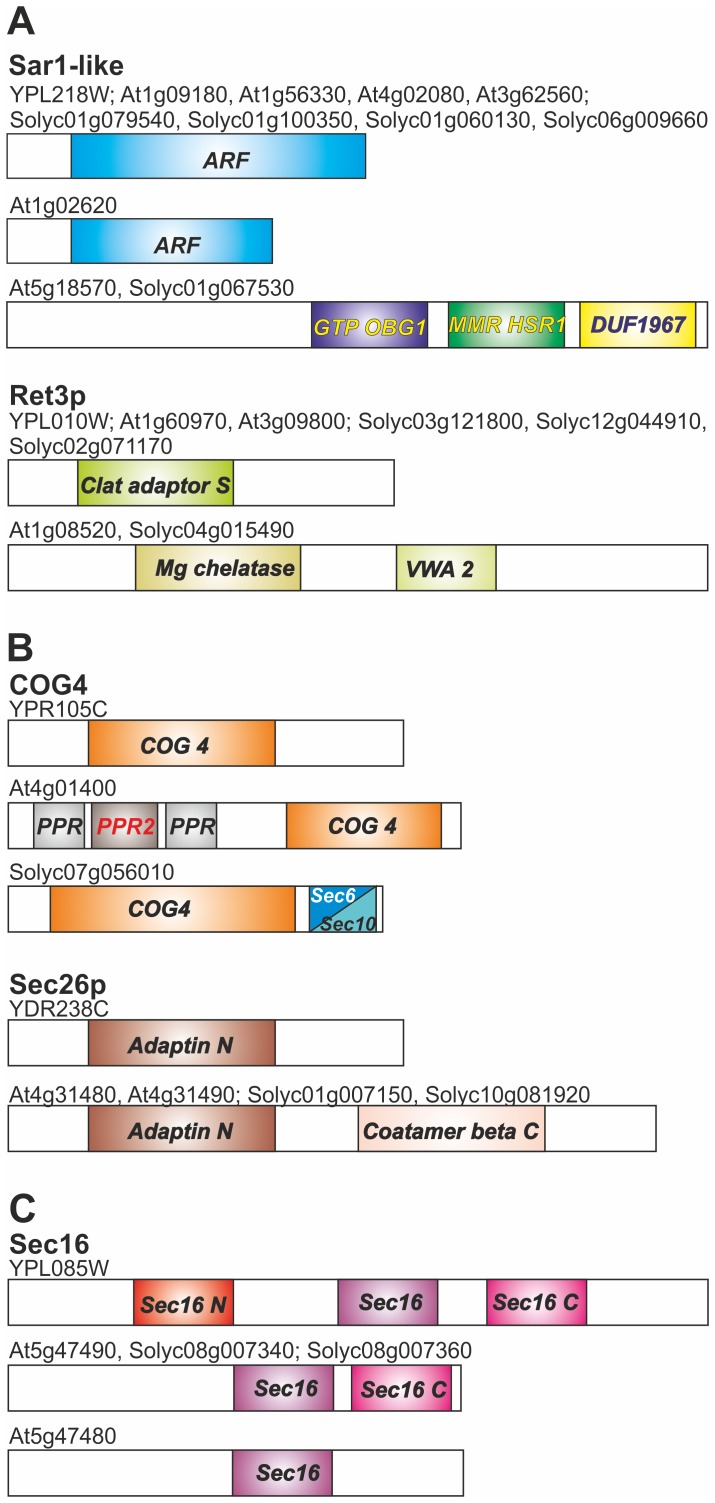
Classification of (co-)orthologues with different domain architecture. (Co-)orthologues of yeast, *A.thaliana* and *S.lycopersicum* belonging to the same orthologous group but with entirely different domain structure (class III) exemplified for Sec17 (A), with additional domains when compared to the bait (class II) exemplified for COG4 and Sec26p (B) and with less domains then the bait Sec16 (C) are represented as bar diagram showing the corresponding domain architectures.

Different domain architectures within the same orthologous groups can be interpreted as gain, loss or swap of functionality of some genes [226]. Further, there are different orthologous groups detected for the same vesicular transport factor, which might be the result of whole genome duplication (WGD) in plants. For some proteins we even find orthologues with entirely different domain structure (class III), like for Sec17, VPS54, SYP61, TYN11 and TYN12 orthologues (Figs. 3a, S2). The (co-)orthologues of the Sec17 protein in yeast, *A. thaliana* and *S. lycopersicum* display different domain architecture from each other.

In some cases, we observed the presence of additional domains in identified orthologues when compared to the bait (class II), e.g. for COG4 or Sec26p (Fig. 3b). COG4 orthologues in all three species contain the COG4 domain (PF08318), while additional domains exist in the N-terminal region of the *A. thaliana* orthologue (At4g01400) and in the C-terminal region of tomato orthologue (Solyc07g056010; Fig. 3b; Table S11, Table S18, Table S26). These additional domains might provide additional regulatory features but do not argue against an involvement of these orthologues in vesicle transport. The same situation is found for the orthologues of Sec26p (Fig. 3b; Table S7, Table S14, Table S22). While the yeast protein contains only an Adaptin N domain (PF01602), the two proteins found to be orthologue in *A. thaliana* and tomato contain an additional Coatamer beta C domain (PF07718) at the C-terminus. Again, this additional domain supports a function in vesicle transport rather than contradicting an involvement in this process.

Finally, in some cases a domain is absent in the identified orthologue (class II) as seen for the Sec16 proteins (Fig. 3c, Table S6, Table S13, Table S21). The yeast Sec16 (YPL085W) contains three domains annotated as Sec16_N (PF12935), Sec16 (PF12932), and Sec16_C (PF12931), while Sec16_N is not present in the (co-)orthologues found in *A. thaliana* and in *S. lycopersicum*. However, Sec16_N appears not to be essential for the function [227] and thus, the one (co-)orthologue in *A. thaliana* (At5g47490) and the found two in *S. lycopersicum* (Solyc08g007340, Solyc08g007360) which possess ‘Sec16’ and ‘Sec16_C’ domains (Fig. 3c) might indeed be involved in vesicle transport. The second *A. thaliana* (co-)orthologue (At5g47480) contains only the ‘Sec16’ domain and thus might be involved in a process distinct from COP-II vesicle transport because the Sec16_C domain is essential for the association of yeast Sec16 to Sec23 [227]. Thus, in case of the absence of domains a manual inspection was needed to judge the involvement of each of the orthologues in vesicle transport. However, in some cases, we find at least two of the above described cases, e.g. SFT11 (Fig. S2, Table S21–S27).

In light of the predicted vesicle transport system in chloroplasts [141], [177], we analyzed the localization of the identified (co-)orthologues in plants by using publically available experimental data (GFP and mass spectrometry data; see Materials and Methods) from SUBA3 [212], FTFLP [213] and PPDB [214] for *A. thaliana*. Moreover, we also looked for the annotation provided by TAIR as well as the evidence in literature concerning localization of specific proteins (Table S3). For (co-)orthologues without experimental confirmed localization, we used a consensus of 20 different localization predictors provided by SUBA3 to assign the presumable localization (see Material and Methods). In parallel, we predicted the localization for the detected (co-)orthologues found in other plant species. However, we limited the number of tools used to 6 programs, which allowed fully automated prediction (Table S4). Consequently, the previous localization studies concerning vesicle transport factors are compared with our approach for chloroplasts (Table 8) and mitochondria (Table 9). Specific factors and characteristics are presented in respective sections below.

**Table 8:** Chloroplast-localized vesicle transport factors.

Family	Name	*A. thaliana* Id	Andersson & Sandelius, 2004 [53]	Khan et al, 2013 [44]	A. thaliana protein localization	Plants with plastid-localized orthologue
COP-II	**Sec13**	**At3g49660**	**Plastid**	**Plastid**	**Plastid** *	**8 of 14**
	*Sec13*	*At2g43770*	*-*	*Plastid*	*Cyto* *	*-*
	**Sec31**	**At1g68690**	**-**	**-**	**Plastid** *	**12 of 14**
	*Sec31*	*At5g38560*	*Plastid*	*Plastid*	*PM^a^*	-
	*Sec31*	*At2g45000*	*Plastid*	*Plastid*	*Nucleus^b^*	-
	**Sec23**	**At4g01810**	**Plastid**	**Plastid**	**Plastid** *	**8 of 14**
	*Sec24*	*At3g44340*	*Plastid*	*Plastid*	*Cyto/PM^c,a^*	-
	*Sec24*	*At4g32640*	*Plastid*	*Plastid*	*Cyto/PM^c,a^*	-
	Sar1-like	At1g09180	-	-	Plastid/PM^d^	2 of 14
	**Sar1-like**	**At5g18570**	**Plastid**	**Plastid**	**Plastid^e^**	**11 of 14**
COP-I	F-COP	At4g34450	-	-	Plastid^f^	7 of 14
	**F-COP**	**At1g08520**	**-**	**-**	**Plastid^g^**	**12 of 14**
	**Sec7-type**	**At4g38200**	**-**	**-**	**Plastid^f^**	**11 of 14**
	**Sec7-type**	**At3g60860**	**-**	**-**	**Plastid** *	**11 of 14**
CCVs	**Heavy chain**	**At3g08530**	**-**	**-**	**Plastid^e^**	**10 of 14**
	Light chain	At2g40060	-	-	PM/Plastid^h^	2 of 14
	**AP4-β4**	**At5g11490**	**-**	**-**	**Plastid** *	**10 of 14**
Rab GTPases	*RABA5e*	*At1g05810*	*Plastid*	*Plastid*	*PM* *	*-*
	RABB1c	At4g35860	-	Plastid	Plastid^f^	2 of 14
	**RABE1b**	**At4g20360**	**-**	**-**	**Plastid^f^**	**11 of 14**
	*RABF1*	*At3g54840*	*-*	*Plastid*	*ER^i^*	*-*
ESCRTs Tetherin-g factor	**Vps5/SNX**	**At5g59190**	**-**	**-**	**Plastid** *	**9 of 14**
	Vps23	At2g38830	-	-	Plastid/Mito*	2 of 14
	*COY1*	*At3g18480*	*-*	*Plastid*	*Golgi^j^*	*-*
	**Exo70**	**At2g39380**	**-**	**Plastid**	**Plastid** *	**6 of 14**
	Exo70	At1g07725	-	Plastid	Plastid*	2 of 14
	Exo70	At2g28650	-	Plastid	Plastid*	2 of 14
	*Exo70*	*At3g55150*	*-*	*Plastid*	*Cyto & Nucleus^k^*	*-*
	*Exo70*	*At5g59730*	*-*	*Plastid*	*Cyto & Nucleus^k^*	*-*
	**COG1**	**At5g16300**	**-**	**Plastid**	**Plastid/Nucleus** *	**5 of 14**
	*COG2*	*At4g24840*	*-*	*Plastid*	*Vacuole^l^*	*-*
	**COG3**	**At1g73430**	**-**	**Plastid**	**Plastid** *	**8 of 14**
	*COG4*	*At4g01400*	*-*	*Plastid*	*Cyto* *	*-*
	**COG5**	**At1g67930**	**-**	**Plastid**	**Plastid** *	**11 of 14**
	*COG6*	*At1g31780*	*-*	*Plastid*	*Cyto^c^*	*-*
	Bet5	At1g51160	-	-	Plastid & Nucelus*	9 of 14
SNAREs	VTI12	At1g26680	-	-	Plastid^f^	2 of 14
	VTI12	At4g31660	-	-	Plastid & Nucleus*	2 of 14
	VTI12	At2g24700	-	-	Plastid*	2 of 14
	VTI12	At4g31690	-	-	Plastid^h^	2 of 14
	*SYP21*	*At5g16830*	*-*	*Plastid*	*Cyto/Golgi^m^*	*-*
	*SNAP33*	*At5g61210*	*-*	*Plastid*	*PM^n^*	*-*
	*VAMP726*	*At1g04760*	*-*	*Plastid*	*PM^o^*	*-*
	*VAMP714*	*At3g24890*	*-*	*-*	*Plastid/Mito* *	*1 of 14*

* Predictions according to our analysis, Cyto- cytoplasm, Mito- mitochondria, PM- plasma membrane; italic indicates genes for which a chloroplast localization is highly questionable; bold indicates genes for which a chloroplasts localization is very likely based on literature evidence and the prediction of chloroplast localized orthologues in many plants; ^a^ Zhang and Peck 2011 [237]; ^b^ Tamura et al. 2010 [283]; ^c^ Ito et al. 2011 [236]; ^d^ Kleffmann et al. 2004 [284] and Mitra et al. 2009 [285]; ^e^ Olinarea et al. 2010 [286] and Garcia et al. 2010 [140]; ^f^ Zybailov et al. 2008 [287]; ^g^ Soldatova et al. 2005 [248]; ^h^ Froehlich et al. 2003 [288]; ^i^ Aker et al. 2006 [289]; ^j^ Latijnhouwers et al. 2007 [167]; ^k^ Chong et al. 2010 [139]; ^l^ Carter et al. 2004 [265]; ^m^ Hummel et al. 2012 [290]; ^n^ Meyer et al. 2009 [291]; ^o^ Uemura et al. 2004 [268].

**Table 9:** Mitochondrial-localized vesicle transport factors

Family	Name	*A. thaliana* Id	Heazlewood et al. 2004 [133]	A. thaliana protein localization	Plants with mitochondrial-localized (co-)orthologue
COP-II	Sec31	At3g63460	Mito	Nucleus/Mito*	1 of 14
COP-I	**B-COP (β)**	**At3g15980**	**Mito**	**Cyto/Mito** *	**9 of 14**
	Sec7-type	At4g35380	Mito	Mito*	2 of 14
	**ARF1A**	**At5g14670**	**Mito**	**Mito** *	**13 of 14**
	**ARF1A**	**At3g62290**	**Mito**	**Cyto/Mito** *	**13 of 14**
CCVs	**σ3**	**At3g50860**	**Mito**	**Cyto/Mito** *	**11 of 14**
	**Υ2**	**At1g47830**	**Mito**	**Mito** *	**11 of 14**
	µ1	At1g60780	Mito	Mito*	6 of 14
	**σ1**	**At2g17380**	**Mito**	**Mito** *	**13 of 14**
	**γ**	**At1g23900**	**Mito**	**Mito** *	**10 of 14**
	**γ**	**At1g60070**	**Mito**	**Mito** *	**10 of 14**
Rab GTPases	**RABG3a**	**At4g09720**	**Mito**	**Mito** *	**10 of 14**
	**SLY1**	**At2g17980**	**Mito**	**Mito** *	**7 of 14**
	RABH1b	At2g44610	Mito	Mito/Cyto/Golgi^a^	4 of 14
	RABH1d	At2g22290	Mito	Mito ^b^	1 of 14
Tethering factors	Exo70	At3g09530	Mito	Mito*	6 of 14
	Exo70	At2g28640	Mito	Mito*	1 of 14
	**COG6**	**At1g31780**	**Mito**	**Mito** *	**11 of 14**

* Predictions according to our analysis, Cyto- cytoplasm, Mito- mitochondria, PM- plasma membrane; bold indicates genes for which a mitochondrial localization is most likely based on Heazlewood et al. [132] and the prediction of mitochondrial localized orthologues in many plants but without further literature evidence; ^a^ Johansen et al. 2009 [292]; ^b^ Heazlewood et al. 2004 [293].

### COP-II-coated vesicles

COP-II vesicles deliver cargo from the site of synthesis at the ER to *cis-*Golgi [16]. Primarily, Sec16 defines the site of assembly of COP-II units [84], [228]. With the exception of *C.reinhardtii* (0), we found 2–5 (co-)orthologues for Sec16 in all the plants analyzed as discussed above (Table S6, Table S13; *A. thaliana*: 2; *S. lycopersicum*: 2).

After assembly site definition, the small G-protein of the Ras superfamily Sar1 is activated by the ER-localized guanine exchange factors (GEF) Sec12 and Sed4 [229], [230]. We observed 1–8 (co-)orthologues for Sar1 (6/5 in *A. thaliana*/*S. lycopersicum*), with one (co-)orthologue (At5g18570) localized in chloroplast as experimentally confirmed (Table 8) [140]. For the GEF factors we observed 2–4 (co-)orthologues in plants (3/1 in *A. thaliana*/*S. lycopersicum*; Table S6, Table S13), but it needs to be mentioned that the orthologues found have a Sec12-like domain architecture (Table1).

The activated Sar1 exposes an N-terminal amphipathic α-helix facilitating its insertion into the membrane and leading to deformation of the ER membrane [231], [232]. Subsequently, Sar1 interacts with the GTPase-activating protein Sec23 to recruit the Sec23–Sec24 heterodimer to form the pre-budding complex [233] in which Sec24 recruits the cargo [234], [235]. In the analyzed plants, we identified up to eight (co-)orthologues for Sec23 (6/4 in *A. thaliana*/*S. lycopersicum*) and for Sec24 (4/4 in *A*. *thaliana*/*S. lycopersicum*; Table S6, Table S13). Interestingly, At4g01810 (Sec23), At3g44340 and At4g32640 (Sec24) are described as putatively chloroplast-localized (Table 8) [141], [177]. By our approach we confirmed the assignment of one Sec23 (co-)orthologue (At4g01810) as plastid-localized, but both Sec24 (co-)orthologues (At3g44340, At4g32640) were assigned to the plasma membrane and cytosol based on experimental evidence (Suba-MS; Table 8, Table S3) [236], [237]. However, one (co-)orthologue of Sec24 in both, *A. thaliana* and tomato (At2g27460 and Solyc11g068500) does not carry the ‘Gelsolin domain (PF00626)’, which is reflected by their smaller protein lengths (Table S13). Further, based on the structural context it is not entirely clear whether this domain is indeed essential for Sec24 function [233].

After formation of the pre-budding complex, an outer coat is formed by Sec13 and Sec31 [43], [238] to shape the membrane for bud formation [239]. We identified 3–10 and 2–9 (co-)orthologues for Sec13 and Sec31 in plants, respectively. Previously, two of the Sec13 (At2g43770, At3g49660) have been assigned as chloroplast proteins [141], [177], while we predict an additional chloroplast-localized protein (At1g68690; Table 8). However, contradicting to the previously described chloroplast localization for At2g43770, we predict cytosolic localization (Table 8). Furthermore, At3g49660 as well as At4g02730 have been described as components of the H3K4 methyltransferase complexes localized in the nucleus [149]. Similarly, one of the (co-)orthologue of Sec13 in yeast (YBR175W) is assigned to perform function in histone methylation [80]. Thus, the orthologue cluster of Sec13 contains proteins involved in two distinct cellular processes.

The Sec31 (co-)orthologues in *A. thaliana* At5g38560 and At2g45000 have been assigned as chloroplast proteins, and we predict a chloroplast localization for the Sec31 (co-)orthologue At1g68690 as well (Table 8) [141], [177]. However, At5g38560 and At2g45000 have been experimental via literature localized to plasma membrane (Suba-MS, TAIR) and nucleus (Suba-GFP, TAIR), respectively (Table 8, Table S3). In line, At5g38560 has been assigned as putative proline-rich extensin-like receptor kinase 8 [111], while At2g45000 was assigned as nuclear pore protein 62 (AtNUP62) [121]. In addition, only At1g18830, At3g63460 and Solyc01g088020 show a similar domain architectures as the yeast bait (Fig. S2, Table 1, Table S21).

In case of *S. lycopersicum*, 3 out of 5 Sec13 and all Sec31 (co-)orthologues are predicted as plastid-localized proteins (Table 8, Table S3), however, in the light of the discrepancy between prediction and experimental evidence for *A. thaliana* Sec31 proteins, the prediction for *S. lycopersicum* Sec31 has to be taken with care.

Finally, the newly configured COP-II vesicle is detached from the ER uncoated by the activity of Sec23 [239] and moves towards the target membrane. For this factor we identified 6 (co-)orthologues in *A. thaliana* and 5 in tomato, all with identical domain structure suggesting that this process involves a multitude of factors in plants (Table 1, Table S21).

### COP-I-coated vesicles

COP-I vesicles mediate the bidirectional transport within the Golgi network (percolating model) [240], [241] and from Golgi apparatus back to the ER [242]. The formation of COP-I vesicles is initiated by the small GTPase of the Ras superfamily Arf1 which in GDP-bound state is adhered to p24 receptors, a group of type-I transmembrane proteins [243]. With the exception of ARF1D, which is only found in *A. thaliana*, we detected orthologues for all Arf1 or Arf-like proteins in all plant species analyzed (Table S7, Table S14) [155]. Further, two ARF1A proteins in *A. thaliana* (At5g14670, At3g62290) are predicted to be mitochondrial-localized (Table 9, Table S3, Table S4), but this prediction is not yet supported by experimental evidence. Similarly, the yeast (YDL137W, YDL192W) and *S. lycopersicum* (Solyc05g005190, Solyc01g008000) (co-)orthologues were also predicted to be mitochondrial-localized as per our analysis (Table S3).

The GEF factors involved in COP-I vesicle transport contains a Sec7 domain and mediate the exchange of Arf1-GDP to Arf1-GTP leading to the exposure of its myristoylated N-terminal amphipathic helix for membrane-anchoring [244], [245]. Subsequently to its activation, *en bloc* recruitment of ‘coatomer unit’ takes place [243]. The ‘coatomer unit’ is composed of two multi-subunit complexes F-COP (cargo selective; β, γ, δ and ζ subunits) and B-COP (cage forming; α, β′ and ε subunits) [246]. All of these coatomer proteins have been identified in plants (Table S7, Table S14). After assembly, COP-I vesicle traverse to the recipient compartment and the Arf1 GTPase-activating protein (ArfGAP) catalyses the Arf1 hydrolysis facilitating the uncoating of the vesicle [3].

In general, (co-)orthologues for all factors of COP-I vesicle have been found in all analyzed plant genomes (Table S7, Table S14) and for ∼60% COP-I (co-)orthologues in *A. thaliana*, experimental evidence (either GFP or mass spectrometry data) for localization exists (Table S3). However, *Z. mays*, *G. max*, *P. patens* and *S. tuberosum* encode higher number of (co-)orthologues for most of the components than other analyzed plants (Table S7). Furthermore, the plant F-COP β (Sec26) and F-COP δ (RET2p) have distinct domain architecture in comparison to the yeast proteins (Table 2). For F-COP β (Sec26), the *A. thaliana* and *S. lycopersicum* proteins possess an additional domain coatamer_beta_c (PF07718) domain that is probably used to regulate the function of N-terminal domain (Adaptin_N: PF01602; Table S22) [247]. In contrast, F-COP δ (RET2p) in *A. thaliana* and *S. lycopersicum* do not contain the clat_adaptor_s (PF01217) domain found in the yeast protein (Table 2, Table S22). One of the identified plant F-COP ζ (RET3; At1g08520) was found in chloroplast [248] and has been described as Mg-chelatase subunit D (CHLD) [194] and thus, is involved in a different cellular process.

Interestingly, except Solyc03g121800, the (co-)orthologues of F-COP ζ in *S. lycopersicum* have also been predicted as plastid-localized (Table S3, Table S4). All identified plant (co-)orthologues for GNOM-type GEF have a ‘Sec7_N’ domain (PF12783), which is absent in the corresponding yeast proteins (YEL022W, YJR031C; Table S22), however, this domain does not argue against their involvement in vesicle transport.

### Clathrin-coated vesicles

Clathrin-coated vesicles (CCVs) deliver cargo from PM and TGN to endosomes [21]. The coatomer of CCVs consists of three light chains bound to three heavy chains, which form a polyhedral lattice [2], [249]. Further, adapter protein (AP) complexes form the ‘cargo-selective’ subunit of CCVs [2]. In general, four AP-complexes are known: AP-1 to -4. The AP-1 complex (γ, β1, µ1 and σ1) functions in vesicle formation at TGN and endosomal compartments, while the AP-2 complex (α, β2, µ2 and σ2) is involved in recruiting cargo proteins from the PM [58], [122], [175], [250]. The AP-3 (δ, β3, µ3, σ3) and AP-4 complex (ε, β4, µ4, σ4) are presumed to play a functional role in TGN-endosomal route and may be associated with clathrin [21].

The components of CCVs have been identified in plants and by manual inspection (Table 3). We did not detect any assigned function distinct from vesicle transport for the proteins in *A. thaliana* and yeast. The factors are by large comparable in their protein length and by the number of (co-)orthologues between *A. thaliana* and *S. lycopersicum* (Table 3, Table S8, Table S15), but we observed certain distinctions in the domain architecture of the plant (co-)orthologues to the yeast factors. For example, one (co-)orthologue of the AP1-µ1 subunit (Solyc04g026830) lacks the ‘Clathrin adaptor complex small chain’ domain (PF01217), while AP2-α subunit (Solyc11g066760) lacks the ‘Adaptin C-terminal’ domain (PF02883) and the ‘alpha adaptin AP2′ domain (PF02296; Table S23), both known to regulate clathrin-bud formation [251]. This poses a high uncertainty for the assignment of the three detected (co-)orthologues as vesicle component.

In contrast, the β1/2′ subunit of AP1 and 2 in plants possess an additional ‘B2-adapt-app C’ (PF09066) and ‘Alpha_adaptin C2’ (PF02883) domain when compared to the yeast protein (Table S23). However, the existence of the latter domain in other yeast proteins YPR029C (ϒ′-AP1) and YBL037W (α-AP2) might compensate for the loss of this domain. Moreover, from the localization analysis, we predicted mitochondrial-localized (co-)orthologues for AP1, AP2 and AP3 factors in yeast, *A. thaliana* and *S. lycopersicum* (Table S3, Table S4). In addition, one (co-)orthologue of heavy chain in *A. thaliana* (At3g08530) was experimentally (FTFLP, PPDB) and via literature (TAIR) localized to plastid and plasma membrane (Table 8, Table S3).

### Retromer and ESCRT complexes

The retromer coat complex possessing a cargo-recognition unit (Vps26, Vps29a and Vps35) and sorting nexins (SNX) are known to recycle the ‘receptor proteins’ back from endosomes [17]. On the contrary, ESCRTs are involved in concentrating and sorting ubiquitinated membrane proteins into invaginations of endosomal membrane (intra-lumenal vesicles ILVs) thereby forming multivesicle body (MVB/s) [252], [253]. Later ILVs release the destined proteins into the vacuole/lysosomes.

We identified orthologues to the described factors in all analyzed plants (Table S9). Analyzing the (co)-orthologues to SNX proteins we realized that all three yeast SNX proteins contain the typical PX domain (PF00787), which is a structural domain involved in phosphoinositide binding and thus in membrane targeting [254], but one of the yeast (YJL036W) as well as a tomato protein (Solyc09g010130) does not carry VPS5 domain (PF09325; Table 4, Table S24). The comparison between (co-)orthologues identified in *A. thaliana* and *S. lycopersicum* manifested that most of the factors have a similar architecture with the exception of SNF7a (Table 4). The latter is exclusively found in *A. thaliana*; as well as VPS2 and VPS31/Bro1, for which the tomato sequences are significantly shorter than the sequences in *A. thaliana* (Table S16, Table S24).

In general, we detected experimental evidence by SUBA3, PPDB or FTFLP or the annotations provided by TAIR for the localization of only 32% Retromer and ESCRT proteins in *A. thaliana* (Table S3). We observed that majority of the identified PIP3P-binding proteins in *A. thaliana* are localized to the cytosol (FTFLP, Suba-MS, PPDB, TAIR) or endosomes (TAIR), wherein the ‘cargo recognition components’ were detected as either cytosolic (FTFLP, PPDB, TAIR) or Golgi localized proteins (TAIR; Table S3). In turn, the majority of the *S. lycopersicum* (co-)orthologues for retromer units were predicted to be cytosolically localized (Table S3). Further, most of the ESCRT components in *A. thaliana* were predicted to be localized to the nucleus, while a few were confirmed via literature or experiments to be localized to the cytosol (At3g12400; TAIR) or plasma membrane (Suba-MS, TAIR) wherein the *S. lycopersicum* (co-)orthologues were predicted as cytosolic, nuclear, mitochondrial and plastidial proteins (Table S3). Again, in the light of the experimental evidence for the *A. thaliana* proteins, the prediction for the *S. lycopersicum* proteins has to be confirmed in future.

### Rab GTPases

Rab GTPases emerge as universal regulators for multiple events ranging from vesicle formation, uncoating and transport to tethering process, and to the final vesicle fusion [255]. GTP bound Rab proteins recruit effector-molecules (e.g. adaptors tethering factors kinases phosphatases and motors) to facilitate vesicle traffic [256], [257]. These proteins have been used as a markers for different compartments; RabB and RabD are known to be localized at Golgi, RabE at ER and Golgi, RabG at vacuoles and RabA to ‘recycling endosomes’ while other Rabs (RabC and RabF) are expected to be localized at endocytic compartments [21], [255].

In line with their importance for the cargo recognition, we identified Rab GTPases belonging to all groups (A to H) in all plant species (Table S10, Table S17). In contrast to the above described factors, we did not identify significant differences in the domain architecture in any of the identified (co-)orthologues. In addition, we did not detect any distinct function proposed for the mentioned *A. thaliana* and yeast (co-)orthologues. While yeast possesses one representative member for majority of the groups, we detected multiple (co-)orthologues in plants (Table S10, Table S17). Most of the classes (A to H) contain ‘Ras domain; PF00071’ typical for small GTPases with the exception of RabE1b, which instead possess domains typical for GTP-binding elongation factor family proteins and the small GTPases containing a ADP Ribosylation Factor type GTPase domain (Table S25). Interestingly, for 66% of the Arabidopsis Rab GTPases, experimental evidence (GFP and mass spectrometry) for their localization is available (Table S3). Moreover, most of the Rab proteins from class A are experimentally known to be localized to trans-Golgi network or endosomes [258], [259], while 3 and 5 proteins from Rab B and E, respectively, are experimentally known to be localized to Golgi or pre-vacuolar compartment (Table S3) [258]. Remarkably, all the Rab proteins are highly conserved in their domain architectures and belong to class I (Table 5).

The Sec1-Munc (SM)-family of proteins which are known to form an association with Qa family of SNAREs [260] are present in all plants as well (Table S10, Table S17) and in almost all cases they contain a so-called ‘Sec domain’ (Sec1 family PF00995; Table S25). This domain generally characterizes proteins involved in vesicle transport processes like exocytosis [261].

### Tethering factors

Tethering factors act upstream of SNAREs to facilitate membrane recognition before fusion [223]. Two types of tethering factors are discussed: homodimeric-tethering factors with elongated coiled-coil regions [262] and multi-subunit tethering complexes (MTCs) [263]. Coiled-coil tethers are long rod-like structures possessing heptad repeats [264]. In yeast, four coiled-coil tethers have been described: Uso1 (p115), COY1 (CASP), RUD3/GRP1 (GMAP210) and Imh1 (Golgin-245) [34], [42], [52], [67], [223]. In plants, we only found orthologues to the homodimeric-tethering factors Uso1 and COY1 (Table S11, Table S18). The two plant (co-)orthologues of Uso1 (At3g27530, Solyc08g081410) are only half the size of their yeast counterpart (YDL058W) but contain the characteristic domains ‘Uso1_p115_head’ and ‘Uso1_p115_C’ (PF04869 and PF04871; Table 6, Table S26), and thus, might indeed be involved in vesicle transport. Interestingly, the COY1 orthologue in *A. thaliana* (At3g18480) is putatively described to be chloroplast-localized [177], but experimental evidence for its localization to Golgi exists (Suba-MS, FFTLP, PPDB; Table 8, Table S3) [160].

Further, four major MTC complexes are discussed. (i) HOPS (homotypic fusion & vacuole protein sorting/class-C vacuole protein sorting (Vps), (ii) an extension of HOPS annotated as CORVET (class C core vacuole/endosome tethering), (iii) the complex associated with tethering containing helical rods (CATCHR) constituting Exocyst, COG, DSL1, and GARP complexes, and (iv) the transport protein particle complex, TRAPP [224].

With the exception of Vps3, all HOPS and CORVET complex components (Vps3, Vps8, Vps11, Vps16, Vps18, Vps33, Vps39 and Vps 41) are found in plants (Table 6). However, Vps39 (HOPS) and Vps8 (CORVET) are class II proteins, which differs slightly in the domain architecture comparing plants and yeast (co-)orthologues (Tabsle 6, Table S11), but are predicted to possess similar localization to that of yeast protein.

The four CATCHR complexes are conserved to a different extent. With the exception of Tip20 (At1g08400) [147], we could not identify orthologues to components of the DSL1 complex (Dsl1 and Sec39; Table S18, Table S26). However, we could not detect (co-)orthologue of Tip20 in tomato, which is detected in other plant species (Table S18). For COGs, (co-)orthologues for all have been identified in both *S. lycopersicum* and *A. thaliana* (Table S18), [159]. Interestingly, 6 COG orthologues in *A. thaliana* have been putatively described to be chloroplast-localized [177], while from our analysis we predicted 3 of 6 (co-)orthologues as plastid-localized (COG1, 3 and 5; Table 8, Table S4), COG4 as cytosolically localized, COG6 as mitochondrial and COG8 as Golgi localized. However, one (COG2, At4g24840) is experimentally confirmed (Suba-MS, TAIR) to have a vacuolar localization (Table 8, Table S3) [265].

Additionally, we identified orthologues to the three GARP components: Vps52 Vps53 and Vps54 but not to Vps51 (YKR020W; Table 6, Table S11). In contrast to the other CATCHR families, we identified orthologues to all eight Exocyst components (Sec3, 5, 6, 8, 10, 15, Exo70 and 84), which also show the same domain architecture as the corresponding yeast protein, except the Sec10 (Table 6, Table S18). For Exo70 we observed a large number of (co-)orthologues in all plant genomes (Table S11, Table S18), however, previous studies showed even larger set of genes representing the Exo70s in *A. thaliana* (23 putative homologues) [131], while we detected only 14 of 23 in the orthologus group corresponding to yeast Exo70. Furthermore, 5 of the 14 Exo70 (co-)orthologues in *A. thaliana* have been predicted as chloroplast-localized [177], while we detected contradictory localization based on experimental evidences (FTFLP, PPDB) for two (co-)orthologues (At3g55150, At5g59730; Table 8) [139]. In addition, we identified three more Exo70 (co-)orthologues (At5g03540, At1g07000, At5g61010) with evidence via experiments and literature to be localized to cytosol, nucleus or plasma membrane (PPDB, FTFLP; Table S3) [139]. Remarkably, with the exception of Trs85 (YDR108W) and Trs65 (YGR166W), other components of TRAPP-I & TRAPP-II complex have been detected in plants (Table 6, Table S18). From the manual inspection, we did not detect any *A. thaliana* or yeast (co-)orthologue to be involved in a process other than vesicle transport.

### SNAREs

SNAREs act as a universal adapter facilitating the fusion of vesicle and recipient compartment. SNARE proteins possess a signature SNARE motif (60–70 amino acids) arranged in heptad repeats which play a role in establishing hetero-oligomeric interactions [19]. Based on the presence of conserved glutamine (Q) or arginine (R) in the center of the SNARE domain, SNAREs are classified into two groups: Q- and R- SNARES [266]. In general, Q-SNAREs (Qa, Qb, Qc, Qb+Qc- SNAREs) are localized on the target compartment whereas R-SNAREs reside on the vesicle [19]. A SNARE complex is composed of four intertwined α-helices; three distinct Q-SNAREs and one R-SNARE [267]. The complex formation enforces a tight association between the opposing membranes thereby initiating the ‘fusion’ event.

In accordance with their reported importance [267], we identified orthologues for almost all SNARE types in both *A. thaliana* and *S. lycopersicum* (Table S19), which are comparable on the basis of protein length and their domain architecture (Table 7, Table S27). Moreover, with few exceptions (Syp112, Pen1, VTI13, SYP61, SYP72, SYP73, and Snap29), we could detect SNARE orthologues in all other plant species as well (Table S12). Further, the plant-specific SNAREs in *A. thaliana*, NPSN (novel plant-specific SNARE) [19], [119] At2g35190, At3g17440 and At1g48240 are experimentally confirmed to be plasma membrane localized (Table S3) [268]. The two (co-)orthologues of NPSN in *S. lycopersicum*, Solyc08g077550 and Solyc12g098950 were predicted to be localized to cytosol, nuclei or Golgi. Furthermore, majority of the Qa SNAREs in *A. thaliana* were experimentally identified in the PM (Table S3) [268]. From the previous studies, SYP21 (At5g16830), SNAP33 (At5g61210), VAMP726 (At1g04760) are putatively described as chloroplast-localized [177], while we detected contradictory localizations compared to the existing experimental evidences (SUBA3, FTFLP, PPDB; Table 8, Table S3) [268].

The detected (co-)orthologues of VTI12, with the exception of At2g24645, At2g24681 and At2g24696, have been described as members of B3 superfamily of proteins and are referred as REM proteins [133], [181]. Only At4g31610 is experimentally characterized as AtREM1, while other (co-)orthologues are putatively classified as REMs [133], [181]. Moreover, At4g00260 has been discussed as MEE45 as well, which plays role in embryo sac development [156]. Thus, considering the existing literature and analyzing the domain architecture (Table 7), at stage it is not clear whether the detected (co-)orthologues of VTI12 have a role in vesicle transport or not.

### Co-regulated clusters of vesicle transport encoding genes

**Figure 4:**
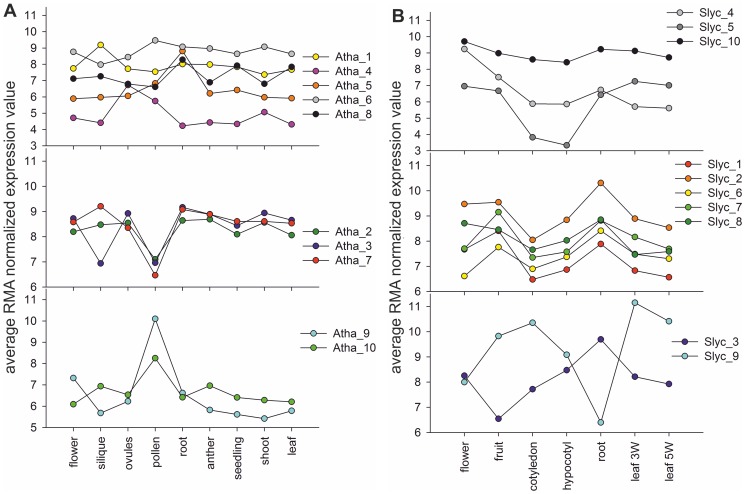
Expression analysis of *A.* thaliana and *S. lycopersicum* genes. Shown are the average RMA normalized expression patterns of different tissues for 10 clusters of vesicle transport factors in (A) *A. thaliana* and (B) *S. lycopersicum*. The y-axis shows the average RMA normalized expression of a maximum of 4 samples per tissue. The 10 clusters (Atha_1 to Atha_10) and (Slyc_1 to Slyc_10) were obtained by k-means clustering and split into four graphs in accordance to their expression profile in different tissues. (Leaf 3W: 3 weeks old leaf; Leaf 5W: 5 weeks old leaf).

Having identified (co-)orthologues for most of the components involved in vesicle transport we aimed at identification of co-regulated clusters of genes. To this end, we used publically available expression data (Table S3, Materials and Methods). The tissue expression data were extracted from the Gene Expression Omnibus (GEO) [269] for 9 and 7 different tissues and developmental stages of *A. thaliana* and *S. lycopersicum,* respectively (Table S5). We performed k-means clustering for both organisms independently. To avoid overweighting of a certain tissue due to a higher number of samples we performed hierarchical clustering of the data for respective tissue to select four representative samples showing a median expression profile. The mean of the tissue samples were further considered as basis for the k-means clustering. The number of clusters (k) was limited to 10 because of the decreased gradient in analyzing the distance to the optimal cluster solution (Fig. S1; Table S28, Table S29). The available data allowed the analysis of 282 of the 340 genes (82%) from *A. thaliana* (Fig. 4a; Table S28) and 149 of 307 *S. lycopersicum* genes (48%, ∼25% of which belongs to Rab GTPases) identified in here as (co-)orthologues of factors involved in the vesicle transport (Fig. 4b; Table S29).

In *A. thaliana,* we detected two clusters (annotated as Atha_9 and Atha_10) of significantly higher expression in mature pollen with respect to other tissues (Fig. 4a). Atha_9 mostly contains orthologues to Rab GTPases and SNARE proteins (32/48) (Table S28), while cluster Atha_10 consists of orthologues to SNARE proteins (18/46), Sec31 (3) and ESCRT components (5; Table S28).

The genes of the clusters Atha_2, Atha_3 and Atha_7 exhibited lower expression in mature pollen, and genes of cluster Atha_3 show reduced expression in siliques (Fig. 4a). The Atha_2 cluster is composed of (co-)orthologues from COP-II (Sec23, Sec24 and Sec13), clathrin units (β4-AP4, β3-AP3, β1/2′-AP1), SNAREs (Qa, R and Qc), Rab GTPases (C, D and E), tethering factors (COG3, COY1, BET3 and BET5). Similarly, Atha_7 represents (co-)orthologues of all COP-II units; Sec13 Sec31, Sec23, Sec24, GEF and GTPase, and Atha_3 exhibits (co-)orthologues of cage and cargo selective units of COP-I vesicle (Table S28).

Genes of the clusters Atha_5 and Atha_8 have enhanced expression in roots or seedlings (in case of Atha_8). In turn, the genes of cluster Atha_1 show the highest expression in siliques, the genes of cluster Atha_4 in ovules and pollen, while the genes of cluster Atha_6 do not show a preferential tissue expression (Fig. 4a). Atha_1 represents (co-)orthologues for ESCRT I (VPS23 VPS37), ESCRT II (VPS36), and ESCRT III (VPS2 DID2) factors, while cluster Atha_5 majorly consists of Rab GTPases and SNARES (11/17; Table S28) in addition to Retromers, ESCRT and clathrin units.

The *A. thaliana* (co-)orthologues to 25 factors are present in different clusters according to their expression pattern. In addition, for 15 factors at least one (co-)orthologue is classified in a distinct cluster, while for four factors all (co-)orthologues were classified in the same cluster (Table S28). Consistently, for *S. lycopersicum* we found 15 factors with all (co-)orthologues in different clusters, 7 factors with at least one (co-)orthologues in a different cluster and only for two factors a classification of all (co-)orthologues in the same cluster (Table S29). Thus, the presence of (co-)orthologues in different clusters strongly suggests possible distinct and overlapping functions.

Comparing the clusters for *A. thaliana* (Fig. 4a, Table S28) with *S. lycopersicum* (Fig. 4b, Table S29), we observed that genes in cluster Slyc_2 and Slyc_3 show an enhanced expression in roots similar as observed for *A. thaliana* co-expression clusters Atha_5 and Atha_8, but not as drastic. Similarly, the Slyc_9 cluster shows an enhanced expression in cotyledon and hypocotyls comparable to the enhanced expression in seedlings for Atha_8. For the clusters Slyc_1, Slyc_6, Slyc_8 and Slyc_10 we do not find a significant alteration of expression, which is comparable to Atha_6. Furthermore, genes of Slyc_7 are higher expressed in fruits, which is comparable to the expression behavior of Atha_1 i.e. expressed more in siliques. In contrast to the *A. thaliana* genes, we found a specific set of genes, which is highly expressed in flowers (Slyc_4) and another set, which has low expression in cotyledon and hypocotyl (Slyc_5). Slyc_4 consists of (co-)rthologues of SNAREs (Sec18, NPSN, SYP21/22/23, GOS12, VAMP, SNAP33) and Rab GTPases (D, E and F), while the Slyc_5 cluster possess (co-)rthologues of SNAREs (Sec22, VAMP, Use11, SFT11/12, MEMB11/12), Rab GTPases (A, B, C, D and G), ESCRTs (Vps37, 2, 25, 22). Unfortunately, for *S. lycopersicum* a large dataset for expression in pollen or ovules is not available. While inspecting the overlap between the genes found in *S. lycopersicum* and *A. thaliana* clusters with similar regulation we did not find a large overlaps with respect to factors assigned with specific pathways, which in part might be explained by the different datasets analyzed. More likely, this might also suggest that evolution has led to the co-regulation of distinct components corresponding to different pathways.

**Figure 5:**
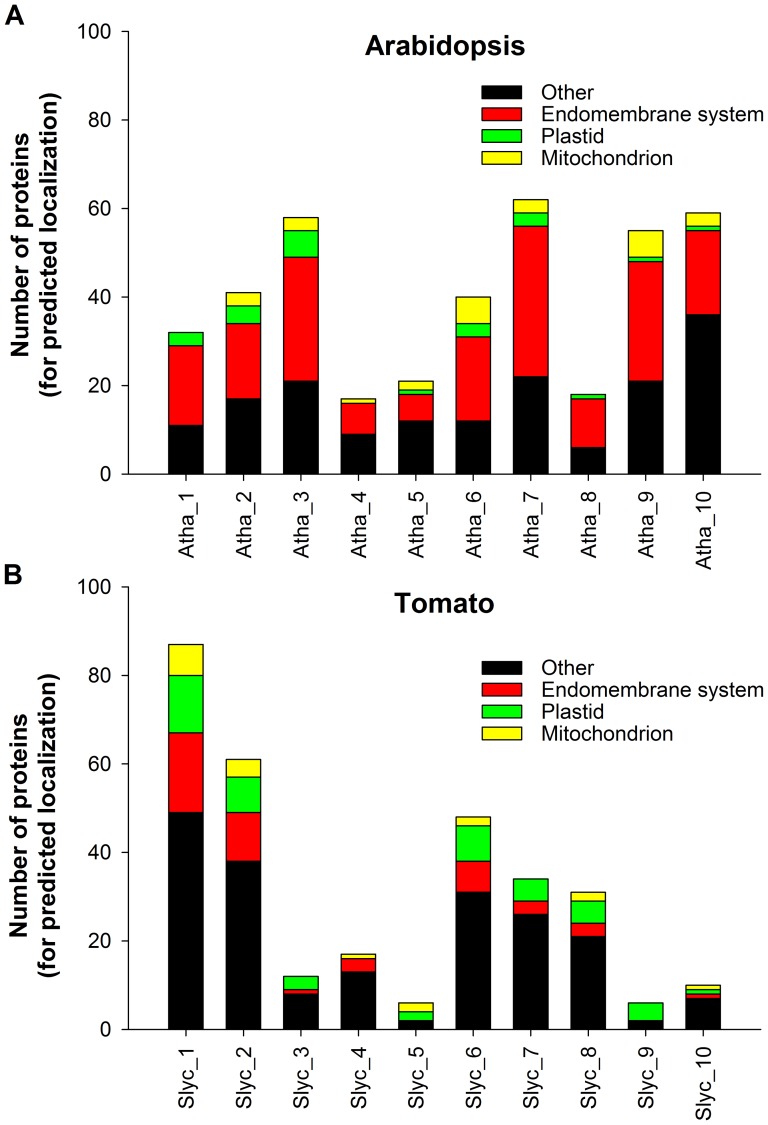
Predicted intracellular localization of factors in different clusters. Shown are stacked bar charts of the factors categorized on the basis of consensus localization analysis. Vesicle transport factors of *A. thaliana* (A) and *S. lycopersicum* (B) are clustered concerning their tissue-specific expression and distributed to their predicted localization. The localization of *A. thaliana* depends on the high certainty approach. For *S. lycopersicu*m, the localization was determined by the low certainty approach (see Materials and Methods).

Further, we analyzed the confirmed or predicted localization of the proteins encoded by the genes of different clusters (Fig. 5). Correlating the localization and expression for specific tissues, no obvious pattern could be observed. Interestingly, *A. thaliana* and *S. lycopersicum* differ in their amount of vesicle proteins localized to specific compartments. For *A. thaliana*, a high amount of plasma membrane localized proteins (represented as endomembrane; Fig. 5) were observed, while in *S. lycopersicum* the localization to plastids and the cytosol was dominating. The latter result might be biased by the large amount of experimentally confirmed localization of *A. thaliana* proteins and only the localization predictions for the *S. lycopersicum* proteins.

## Discussion

### The complexity of the vesicle transport system in plants

We identified (co-)orthologues of components involved in vesicle transport in 14 plant species and yeast (Fig. 2; Table S6–S12). These (co-)orthologues were based on the ‘core-set’ of 240 factors extracted from literature in yeast or *A. thaliana* (Fig. 1). In yeast, (co-)orthologues for 171 factors were identified (Tables 1–7). However, this is reflected by 129 (co-)orthologues only, as some of the factors belong to the same orthologous group. In addition, with the exception of one (co-)orthologue for Sec13, all (co-)orthologues have been assigned to class I suggesting an involvement in vesicle transport. For the 69 remaining factors described to be involved in vesicle transport in *A. thaliana*, orthologues do not exist in yeast, namely for Rab GTPases (29; Table 5, Table S17), SNAREs (26; Table 7, Table S19), COP-I vesicles, CCVs (5 each; Table 2, Table 3, Table S14, Table S15) and ESCRTs (4; Table 4, Table S16).

In the 14 plant genomes a total of 4021 (co-)orthologous sequences corresponding to the ‘core-set’ of factors are identified. Only 8 tethering factors found in yeast are not observed in plants (Table 6, Table S18); namely Rud3/Grp1 and Imh1 (coiled coils), Vps3 (CORVET), Dsl1 and Sec39 (DSL1 complex), Vps51 (GARP complex), as well as Trs85 and Trs65 (TRAPP-I and II). The highest number of genes per factor is present in *G. max* (481; ∼2 genes/factor) and *P. trichocarpa* (341; >1 genes/factor). This may be credited to recent whole genome duplications (WGD) [270], [271]. It might be speculated that the time that has passed after WGD was not sufficient to deselect redundant factors of duplicated regions. The lowest number of (co-)orthologues is obtained for *C. reinhardtii* (118; <1 genes/factor), which is consistent with its small genome size. Moreover, the number of (co-)orthologues is relatively constant in all monocots with the exception of *Z. mays.* The latter reflects that *Z. mays* is the only investigated monocot with recent whole genome duplication [272]. In contrast, the analyzed dicots show a higher variation in the number of identified (co-)orthologues of the vesicle transport factors as several dicots had a recent whole genome dupli-/triplications (Table S20).

Species specificities in the proteome of vesicle transport factor exist as well. For example, no (co-)orthologue for Sec16 (COP-II, Table S6) or the light chain of the triskelion and AP3 complex is found in *C. reinhardtii* (CCVs; Table S8). Similarly, *S. tuberosum* and *M. truncatula* do not possess light chains of the triskelion (Table S8). However, it cannot be excluded that the absence of factors might result from incomplete gene annotation of the respective genomes and should be taken with care. Generally, plants appear to show a higher complexity with respect to the vesicle transport when compared to yeast (Table S6–12, Table S20). This on the one hand results from the existence of chloroplast as additional organelle and on the other hand most likely reflects certain tissue specificity in the expression pattern of individual genes.

With respect to the ‘core-set’ of 240 factors, 340 (co-)orthologues in *A. thaliana* and 307 in *S. lycopersicum* are assigned to orthologous groups (Tables 1–7, Table S13–S19), 275 *A. thaliana* and 232 *S. lycopersicum* protein sequences are of class I, and thus most likely involved in vesicle transport. The difference in the number of (co-)orthologues is mainly accounted by Rab GTPase (∼4.5 times more in *A. thaliana* and tomato, Table S17) and SNARES (3–4 times more in *A. thaliana* and tomato, Table S19). Furthermore, 149 of the 212 factors described for *A. thaliana* have been identified in all plants. Interestingly, 7 factors are specifically found only in *A. thaliana*, namely ARF1D, RabA4b, Raba4e, Rabc2b, RabG1, KEULLE and SYP24 (Table 1–7).

### Tissue-specificity of vesicle factors in *A. thaliana* and *S. lycopersicum*

Clustering of the available tissue specific expression studies based on *S. lycopersicum* GeneChip (9,200 transcripts) containing 149 of the 307 different (co-)orthologues for vesicle transport revealed a comparable behavior for most genes. In general, the clusters showed a higher expression in roots, flowers and fruits when compared to hypocotyl and cotyledon tissues and an intermediate expression in leafs (Fig. 4b). Only two clusters show a significantly different expression, namely low expression in fruits (cluster Slyc_3), which is only represented by one RABH1 orthologue (Table S29), or high expression in hypocotyl and cotyledon and low expression in roots (cluster Slyc_9). Again, this cluster represents only 3 orthologues, one to RABE1a, one to RABE1b and one to Arl8-like. Thus, our analysis only presents a first indication, but for a final conclusion more experimental data are required.

In contrast, the publically available expression data for *A. thaliana* genes is sufficient to justify conclusions (Fig. 4a). Remarkably, (co-)orthologues for all components of COP-II vesicle are represented in cluster Atha_7, which display a high expression in all tissues except of pollen (Fig. 4a, Table S28). This might suggest that a pollen-specific COP-II composition exists, which is supported by the clustering of one Sec16, one Sec13, one Sar1-like and one Sec31 (class II) orthologue in Atha_9/Atha_10, which represent cluster with high expression exclusively in pollen. In turn, cluster Atha_9/Atha_10 contains genes coding for orthologues of all inspected complexes except of CCV-transport factors (Fig. 4a, Table S28). This suggests that the pollen specificity of vesicle transport is rather defined by specific expression of RAB and SNARE genes, of which about 35% of all found (co-)orthologous genes are present in cluster Atha_9/Atha_10.

Atha_5 is the only other cluster which unifies a set of genes with a tissue specific expression, namely with highest expression in roots (Fig. 4a, Table S28). The analysis documents that no orthologues to COP-I, COP-II and CCV factors (with the exception of µ1-AP1) is specifically expressed in roots, while most of the genes found in this cluster are RAB orthologues. Thus, while pollen specific-expressed orthologues exist for many components, roots do not represent a tissue with a large specific set of vesicle transport factors.

### Vesicle transport systems in both chloroplasts and mitochondria?

**Figure 6:**
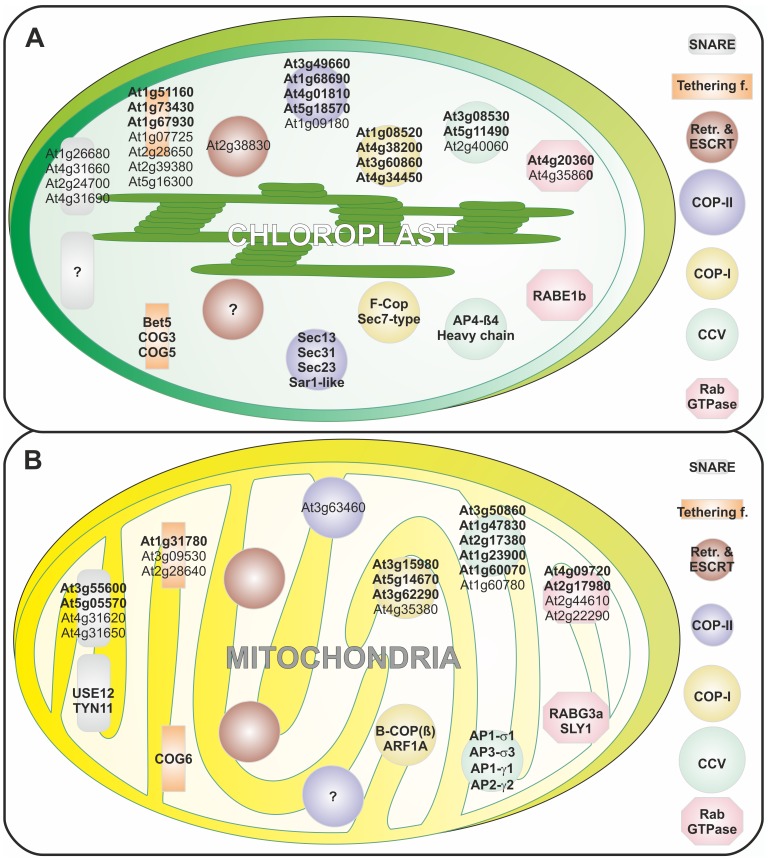
Putative chloroplast or mitochondrial localized vesicle transport factors. Shown are the likely (co-)orthologues of *A. thaliana* (top) and the most likely factors based on the analysis of all 14 plant genomes (present in more then 7 plant genomes, bottom) which are predicted to be chloroplast (A) or mitochondrial (B) localized. The (co-)orthologues are assigned concerning the seven different vesicle transport factor families. The size of the symbol on the left size indicates the importance of the factor family.

Based on bioinformatics approaches, a vesicle transport system has been discussed to be present inside the chloroplasts [141], [177]. This analysis was extended here by utilizing experimental evidences, a multitude of prediction server and localization prediction for orthologues from all plants analyzed (Fig. 6, Table S4). Thus, while 26 factors in *A. thaliana* were previously proposed to be involved in chloroplast-localized vesicle transport system [141], [177], we predict chloroplast localization for 15 proteins in plants, namely four COP-II (Sec13, Sec31, Sec23 and Sar1-like), three for COP-I (F-COP and Sec7-type), two CCVs (Heavy chain and AP4-β4), one RAB GTPase (RABE1) and vive tethering factor components (Vps5, Exo70, COG1, COG3 and COG5; Table 8). From this result it is tempting to speculate that the chloroplast vesicle transport system is similar the COP-II system for transport from ER to Golgi. Nevertheless, the presented large-scale analysis supports the previous proposal of a chloroplast intrinsic vesicle transport system [141], [177]. However, for most of the factors the chloroplast localization has to be experimentally confirmed, particularly for the central components of the vesicles; the cage and cargo-selective units.

Unexpectedly, we also realized (co-)orthologues for which a mitochondrial localization is predicted or even experimentally confirmed (Table 9). However, in contrast to the chloroplast inventory which is dominated by COP-II components and tethering factors, most of the proteins predicted to be mitochondrial localized are (co-)orthologues for CCVs components (Fig. 6, Table S3, Table S4). Approaching the MitoMiner database [273], we observed experimental evidence based on GFP tagging or mass spectrometry for mitochondrial localization in yeast for some (co-)orthologues of Rab GTPases, tethering factors and CCV component as well. Nevertheless, the mitochondrial localization of these factors has not been discussed till date. If one considers that (i) a third of yeast mitochondrial proteome shows dual localization and (ii) that proteins with dual localization have a weaker mitochondrial targeting signal [274], it is possible that at least some of these proteins are indeed mitochondrial-localized.

At stage, a mitochondrial vesicle transport system has not been described. However, at a theoretical level, vesicle-like structures have been proposed to be involved in cristae formation [275]. The ‘Cristae fission–fusion’ model suggests that transiently formed vesicles are implicated in the propagation of the cristae membranes, through budding off from pre-existing cristae and fusion with the inner membrane at different site [275]. Consistent with this idea, Mulkidjanian et al. [276] suggested that the intracellular vesicles of purple bacteria like *Rhodobacter capsulatus* (e. g. Borghese et al. [277]) discussed as close relative to the ancestral endosymbiont leading to mitochondria [278] are the evolutionary precursor of cristae. In line, mitochondrion internal vesicle-like structures have been reported in mitochondria of patients with defective gene functions which cause pathological conditions, or during reconstruction of the matrix compartment after extensive osmotic swelling [279], [280] as well as in degenerating mitochondria in vascular bundle in petals of open Dendrobium cv. Lucky Duan flowers [281]. Therefore, one can speculate that some of the components identified in this study as mitochondrial-localized factors are involved in the formation of cristae as the induction of membrane curvature is comparable to vesicle formation [282]. Nevertheless, the exact need for mitochondrial-localized vesicle transport factor remains elusive and is subject to verification.

## Supporting Information

Figure S1 Determination of number of clusters (k) for k-means clustering. Shown are the distances of the clustering from the optimal solution (dividing each factor to a single cluster) using enumerated amount of clusters. The k-means clustering is performed for *A. thaliana* (black dot) and *S. lycopersicum* (white dot) using 1 to 50 clusters. The red dashed line marks the number of clusters used for the clustering in this study where the logarithmic distance to the optimal solution has a decreased slope.
(TIF)

Figure S2 Domain architecture of different classes. Shown are the domain architecture of (co-)orthologues within one orthologous group for the factors (a) Ret3p, (b) Sec26p, (c) Sec31, (D) SFT11 in yeast, *A. thaliana* and *S. lycopersicum*.
(TIF)

Table S1 Literature reference for all the yeast (co-)orthologues for the vesicular transport factors from the SGD.
(XLSX)

Table S2 Literature reference for all the Arabidopsis (co-)orthologues for the vesicular transport factors from the TAIR.
(XLS)

Table S3 Localization analysis for yeast, Arabidopsis, and tomato (co-orthologues) for COP-II components. For yeast and tomato, Yloc, WoLF PSORT, MitoPred, ChloroP, Target P, Predotar predictors were used and the consensus was built. The score given (for e.g. 1 of 2 or 2 of 3) refers to the prediction given by ‘X’ of the ‘Y’ predictors. In contrast, for Arabidopsis publically available experimental data; GFP (green fluorescent protein) localization/mass spectrometry (SUBA3, FTFLP, PPDB) were utilised. We also looked into the annotation given by TAIR database with a provided reference (PMID). Further, if no experimental evidence existed, we used the consensus of 20 different predictors to assign the probable localizations (as in SUBA3) and the score is presented respectively (for e.g. 11/19 or 5/14 etc.). Highlighted cells signify experimental evidence for the particular (co-)orthologue.PM: plasma membrane, VACU: vacuole, PLAS: plastid, MITO: mitochondria, NUCL: nucleus; CYTO: cyoplasm, GOLG: golgi, ER: endoplasmic reticulum, PERO: peroxisome, EX-CE: extra-cellular, CY-SK: cytoskeleton. The experimentally proven localisations are highlighted.
(XLSX)

Table S4 Localization analysis of chloroplast or mitochondrial localized (co-)orthologues in other analysed plant species in context of chloroplast or mitochondrial-localized *A. thaliana* (co-)orthologues. The highlighted cells shows that the respective factor has 7 or >7 of the 14 plant species possessing the similar localization. Except *A. thaliana*, Yloc, WoLF PSORT, MitoPred, ChloroP, Target P, Predotar predictors were used to build a consensus for all other plant species.
(XLSX)

Table S5 GEO IDs considered for downloading microarray data for both *A. thaliana* and *S. lycopersicum* for clustering analysis.
(XLSX)

Table S6 The orthologues of COP-II components identified via OrthoMCL in all the species discussed.
(XLSX)

Table S7 The orthologues of COP-I components identified via OrthoMCL in all the species discussed.
(XLSX)

Table S8 The orthologues of Clathrin coated vesicles components identified via OrthoMCL in all the species discussed.
(XLSX)

Table S9 The orthologues of retromer and ESCRT components identified via OrthoMCL in all the species discussed.
(XLSX)

Table S10 The orthologues of Rab GTPases components identified via OrthoMCL in all the species discussed.
(XLSX)

Table S11 The orthologues of tethering factors components identified via OrthoMCL in all the species discussed.
(XLSX)

Table S12 The orthologues of SNARE components identified via OrthoMCL in all the species discussed.
(XLSX)

Table S13 The COP-II-coated vesicle components of yeast, *A. thaliana* and tomato identified via OrthoMCL and PGAP.
(DOCX)

Table S14 The COP-I-coated vesicle components of yeast, *A. thaliana* and tomato identified via OrthoMCL and PGAP.
(DOCX)

Table S15 The Clathrin-Coated Vesicle (CCVs) transport factors of yeast, *A. thaliana* and tomato identified via OrthoMCL and PGAP.
(DOCX)

Table S16 The Retromer and ESCRT transport factors of yeast, *A. thaliana* and tomato identified via OrthoMCL and PGAP.
(DOCX)

Table S17 The Rab GTPase components of yeast, *A. thaliana* and tomato identified via OrthoMCL and PGAP.
(DOCX)

Table S18 The Tethering factors of yeast, *A. thaliana* and tomato identified via OrthoMCL and PGAP.
(DOCX)

Table S19 The Q and R-SNARE components of yeast, *A. thaliana* and tomato identified via OrthoMCL and PGAP.
(DOCX)

Table S20 ’Core-set’ of plant orthologues for vesicle transport factors.
(XLSX)

Table S21 Domain architecture of components of COP-II coated vesicles in Yeast, *A. thaliana* and *S. lycopersicum*.
(XLS)

Table S22 Domain architecture of COP-I components in yeast, *A. thaliana* and *S. lycopersicum*.
(XLS)

Table S23 Domain architecture of clathrin coated vesicle components in yeast, *A. thaliana* and *S. lycopersicum*.
(XLS)

Table S24 Domain architecture of retromer and ESCRT components in yeast, *A. thaliana* and *S. lycopersicum*.
(XLS)

Table S25 Domain architecture of RabGTPase components in yeast, *A. thaliana* and *S. lycopersicum*.
(XLS)

Table S26 Domain architecture of tethering factors in yeast, *A. thaliana* and *S. lycopersicum*.
(XLS)

Table S27 Domain architecture of SNARE components in yeast, *A. thaliana* and *S. lycopersicum*.
(XLS)

Table S28 *A. thaliana* genes with their description sorted according to the clusters.
(XLSX)

Table S29 *S. lycopersicum* genes with their description sorted according to the clusters.
(XLSX)
