# Supplementary figures and images for: The Complexity of Vesicle Transport Factors in Plants Examined by Orthology Search
Source: PLoS One. 2014 May 20;9(5):e97745. doi: 10.1371/journal.pone.0097745 (PMC4028247; doi:10.1371/journal.pone.0097745)

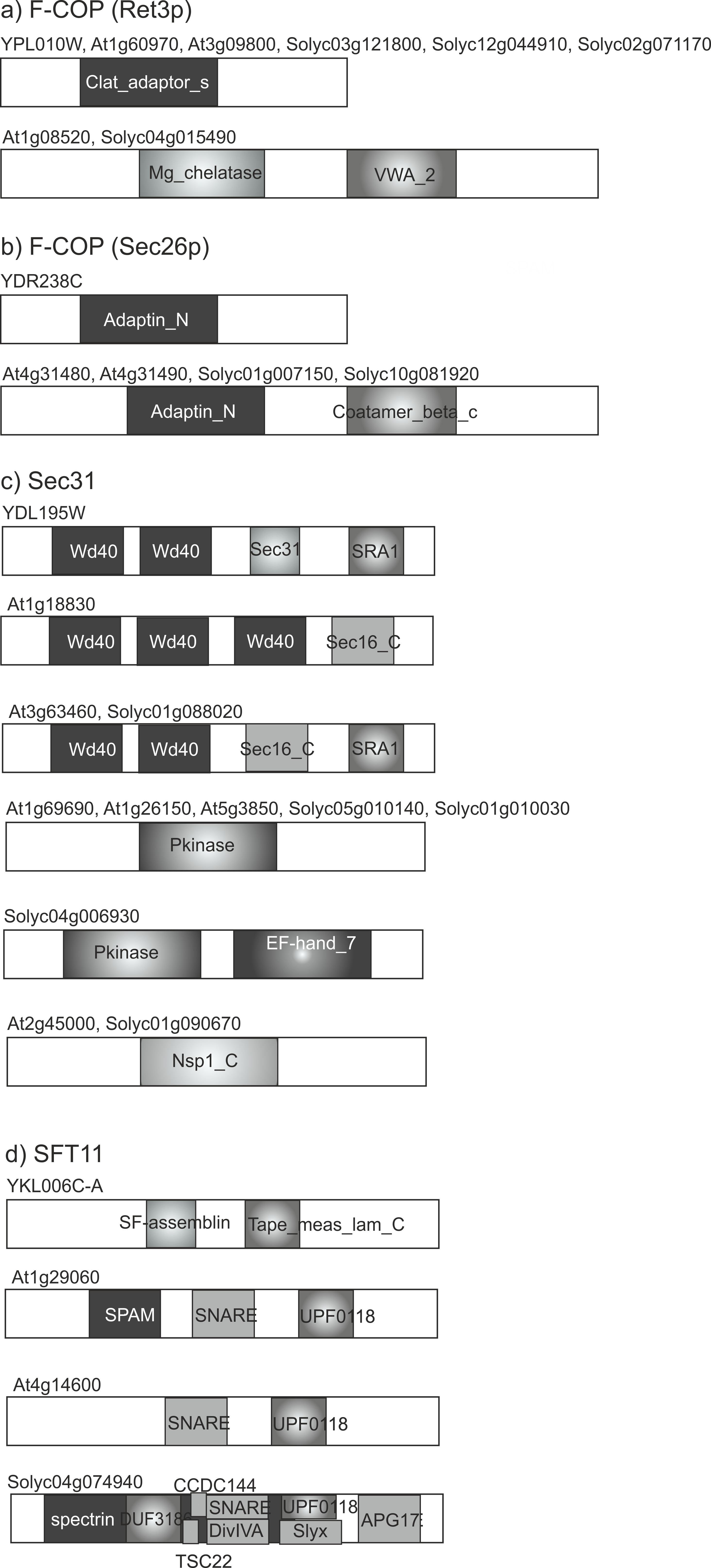

Supplement: Figure S2 — Domain architecture of different classes. Shown are the domain architecture of (co-)orthologues within one orthologous group for the factors (a) Ret3p, (b) Sec26p, (c) Sec31, (D) SFT11 in yeast, A. thaliana and S. lycopersicum. (TIF) [file pone.0097745.s002.tif]
