# Supplementary material for: The Complexity of Vesicle Transport Factors in Plants Examined by Orthology Search
Source: PLoS One. 2014 May 20;9(5):e97745. doi: 10.1371/journal.pone.0097745 (PMC4028247; doi:10.1371/journal.pone.0097745)
Supplement: Table S13 — The COP-II-coated vesicle components of yeast, A. thaliana and tomato identified via OrthoMCL and PGAP. (DOCX) [file pone.0097745.s015.docx]

| **Table S7:**  The COP-II-coated vesicle components of *A. thaliana* and tomato | | | | | |
| --- | --- | --- | --- | --- | --- |
| **Com.** | **Fac.** | **Yeast** | ***A. thaliana*** | ***S. lycopersicum*** | |
| Coatomer  Sec13/31 (cage) | Sec13 | YLR208W(297);YBR175W (315)^ | At2g30050(302); At3g01340(302); At3g49660(317)^; At4g02730(333)^ | Solyc02g087300(302); Solyc02g091790(314); Solyc03g120900(301); Solyc09g065290(315) | |
|  |  |  | *At2g43770(343)* | Solyc03g059100(326) | |
|  | Sec31 | YDL195W(1273) | At1g18830(969); At3g63460(1104) | Solyc01g088020(1124) | |
|  |  | YJL041W(823)^ | At2g45000(739)^ | Solyc01g090670(759) | |
|  |  | NF | At1g26150(762); At1g68690(708);  At5g38560(681) | Solyc01g010030(730); Solyc04g006930(800); Solyc05g010140(750) | |
| Coatomer  Sec23/24 (cargo selective) | Sec23 | YPR181C(768) | *At1g05520(783); At2g21630(761);*  *At3g23660(765); At4g14160(621); At5g43670(794)* | Solyc01g081070(764); Solyc03g005380(778); Solyc05g053830(784); Solyc09g092680(766) | |
|  |  | NF | *At4g01810(880)* | Solyc01g080850(875) | |
|  | Sec24 | YIL109C(926)  YNL049C(876) | At3g07100(1038) | Solyc02g082220(1030) | |
|  |  | NF | At3g44340(1096); *At4g32640(1080)* | Solyc05g055690(1091); Solyc06g084210(1069) | |
|  |  | NF | *At2g27460(745)* | Solyc11g068500(743) | |
| GEF | Sec12 & Sed4 | YNR026C(471);  YCR067C(1065) | At2g01470(393); At5g50550(383); At5g50650(383) | Solyc07g047970(391) |  |
| GTPase | Sar1-like | YPL218W(190) | At1g56330(193); At1g09180(193);  *At3g62560(193)*; At4g02080(193) | Solyc01g060130(194); Solyc01g079540(194);  Solyc01g100350(194); Solyc06g009660(194) | |
|  |  | NF | At5g18570(681) | Solyc01g067530(671) | |
|  |  | NF | *At1g02620(122)* | NF | |
| Misc | Sec16 | NF | At5g47480(1350); At5g47490(1361) | Solyc08g007340(1426); Solyc08g007360(1470) | |
|  |  | YPL085W(2195) | NF | NF | |
| Given are the names of the complex, the name used for the factor in yeast, the gene accession number and in brackets the amino acid length of the (co-)orthologues in yeast, *A. thaliana* and *S. lycopersicum*. Underlined accession Ids were used as bait to identify orthologues, accession Ids in italics are bioinformatically identified as per previous studies  Misc.: miscellaneous, ^discussed to possess function distinct than vesicle transport | | | | | |
