# Supplementary material for: The Complexity of Vesicle Transport Factors in Plants Examined by Orthology Search
Source: PLoS One. 2014 May 20;9(5):e97745. doi: 10.1371/journal.pone.0097745 (PMC4028247; doi:10.1371/journal.pone.0097745)
Supplement: Table S14 — The COP-I-coated vesicle components of yeast, A. thaliana and tomato identified via OrthoMCL and PGAP. (DOCX) [file pone.0097745.s016.docx]

| **Table S10:** The COP-I-coated vesicle components of *A. thaliana* and tomato | | | | | | |
| --- | --- | --- | --- | --- | --- | --- |
| **Com.** | **Factor** | **Yeast** | | ***A. thaliana*** | ***S. lycopersicum*** | |
| B-COP (cage) | α (RET1p) | YDL145C(1201) | *At1g62020(1216)*; *At2g21390(1218)* | | Solyc04g080980(1218); Solyc07g032100(1219) | |
|  | β' (Sec27p) | YGL137W(889) | *At1g52360(889)*; *At3g15980(930)*;  *At1g79990(1135)* | | Solyc03g120270(917); Solyc06g066730(916);  Solyc12g043000(925) | |
|  | ε (Sec28p) | YIL076W(296) | *At2g34840(296)*; *At1g30630(292)* | | Solyc02g030250(291) | |
| F-COP (cargo selective) | β (Sec26p) | YDR238C(973) | *At4g31480(948)*; *At4g31490(948)* | | Solyc01g007150(949); Solyc10g081920(911) | |
|  | Υ (Sec21p) | YNL287W(935) | At4g34450(886) | | Solyc01g109540(886) | |
|  | δ (RET2p) | YFR051C(546) | *At5g05010(546)* | | Solyc01g103480(527); Solyc10g038120(527) | |
|  | ζ (RET3p) | YPL010W(189) | At4g08520(181); *At1g60970(177)*; *At3g09800(179)* | | Solyc02g071170(177); Solyc03g121800(184);  Solyc12g044910(183) | |
| GEF | Sec7-type | YDR170C(2009) | At1g01960(1750); At3g43300(1758)  At3g60860(1793); At4g35380(1706)  At4g38200(1687) | | Solyc01g091460(1779); Solyc12g017830(1771);  Solyc01g112350(1373) | |
|  | GNOM-type | YEL022W(1459)  YJR031C(1408) | At1g13980(1451); At5g19610(1375)  At5g39500(1443) | | Solyc02g067010(1448); Solyc02g088290(1410); Solyc03g070470(1532); Solyc04g005560(1443); Solyc05g013040(1449) | |
| GTPases  ARF | ARF1A | YDL137W(181)  YDL192W(181) | *At1g10630(181)*; At1g23490(181); *At1g70490(181)*; At2g47170(181);  *At3g62290(181)*; *At5g14670(188)* | | Solyc01g008000(209); Solyc01g100860(181);  Solyc02g092970(191); Solyc05g005190(181) | |
|  | ARF1B | NF | *At2g15310(205)* | | NF | |
|  |  | NF | *At3g03120(192)*; *At5g17060(192)* | | Solyc03g096910(132); Solyc06g072800(132) | |
|  | ARF1C | NF | *At3g22950(183)* | | Solyc03g005300(183) | |
|  | ARF1D | NF | *At1g02430(190)*; *At1g02440(190)* | | NF |  |
| GTPases  ARF-like | ARLA | NF | *At3g49870(184)*; *At5g37680(184)*; *At5g67560(184)* | | Solyc02g064640(184); Solyc02g092330(184); Solyc03g043960(184); Solyc11g011040(184) | |
|  |  | NF | *At3g49860(176)* | | NF | |
|  | ARLB | YPL051W(198) | *At5g52210(205)* | | Solyc10g074630(193) | |
|  | ARLC | NF | At2g18390(185) | | Solyc07g014650(169) | |
| Given are the names of the complex, the name used for the factor in yeast, the gene accession number and in brackets the amino acid length of the (co-)orthologues in yeast, *A. thaliana* and *S. lycopersicum*. Underlined accession Ids were used as bait to identify orthologues, accession Ids in italics are bioinformatically identified as per previous studies | | | | | | |
