# Supplementary material for: The Complexity of Vesicle Transport Factors in Plants Examined by Orthology Search
Source: PLoS One. 2014 May 20;9(5):e97745. doi: 10.1371/journal.pone.0097745 (PMC4028247; doi:10.1371/journal.pone.0097745)
Supplement: Table S15 — The Clathrin-Coated Vesicle (CCVs) transport factors of yeast, A. thaliana and tomato identified via OrthoMCL and PGAP. (DOCX) [file pone.0097745.s017.docx]

| **Table S13:** The Clathrin-Coated Vesicle (CCVs) transport factors of *A. thaliana* and tomato | | | | | |
| --- | --- | --- | --- | --- | --- |
| **Com.** | **Factor** | **Yeast** | | ***A. thaliana*** | ***S. lycopersicum*** |
| Triskelion (cage) | Heavy chain | YGL206C(1653) | At3g08530(1703); At3g11130(1705) | | Solyc03g096000(1702); Solyc05g052510(1706)  Solyc06g051310(1701) |
|  | Light chain | NF | At2g40060(258); At3g51890(258) | | Solyc09g014450(236) |
|  |  | YGR167W(233) | NF | | NF |
| AP1 | γ | YPR029C(832) | At1g23900(876); At1g60070(898) | | Solyc04g025870(877); Solyc05g005780(879) |
|  | β1 & β 2' | YKL135C(726) | *At4g11380(916)*; *At4g23460(893)* | | Solyc08g081320(896) |
|  | μ1 | YPL259C(475) | At1g10730(428); At1g60780(428) | | Solyc04g026380(235); Solyc05g013340(426); Solyc06g071650(416) |
|  | σ1 | YLR170C(156) | At2g17380(161); *At4g35410(162)* | | Solyc11g005220(161) |
| AP2 | α | YBL037W(1025) | At5g22770(1012); At5g22780(1013) | | Solyc06g074650(1017); Solyc11g066760(468); Solyc11g066770(466) |
|  | μ2 | NF | At5g46630(441) | | Solyc06g061150(439); Solyc08g006960(438) |
|  | σ2 | YJR058C(147) | *At1g47830(142)* | | Solyc04g081410(142) |
| AP3 | δ | YPL195W(932) | At1g48760(869) | | Solyc03g119270(970) |
|  | β3 | YGR261C(809) | At3g55480(1115) | | Solyc06g043030(1318) |
|  | μ3 | NF | *At1g56590(415)* | | Solyc01g100820(415) |
|  | σ3 | YJL024C(109) | *At3g50860(194)* | | Solyc01g109640(166); Solyc02g085240(167) |
| AP4 | ε | NF | *At1g31730(938)* | | Solyc08g075310(968) |
|  | β4 | NF | *At5g11490(850)* | | Solyc08g068210(840) |
|  | μ4 & σ4 | NF | At4g24550(451) | | Solyc04g076230(452) |
| Given are the names of the complex, the name used for the factor in yeast, the gene accession number and in brackets the amino acid length of the (co-)orthologues in yeast, *A. thaliana* and *S. lycopersicum*. Underlined accession Ids were used as bait to identify orthologues, accession Ids in italics are bioinformatically identified as per previous studies | | | | | |
