# Supplementary material for: The Complexity of Vesicle Transport Factors in Plants Examined by Orthology Search
Source: PLoS One. 2014 May 20;9(5):e97745. doi: 10.1371/journal.pone.0097745 (PMC4028247; doi:10.1371/journal.pone.0097745)
Supplement: Table S16 — The Retromer and ESCRT transport factors of yeast, A. thaliana and tomato identified via OrthoMCL and PGAP. (DOCX) [file pone.0097745.s018.docx]

| **Table S16.** The Retromer and ESCRT transport factors of *A. thaliana* and tomato | | | | |
| --- | --- | --- | --- | --- |
| **Com** | **Factor** | **Yeast** | ***A. thaliana*** | ***S. lycopersicum*** |
| PIP3P-binding | Vps5/  SNX | YJL036W(423)  YKR078W(585)  YOR069W(675) | At5g06140(402); At5g07120(572);  At5g58440(587) | Solyc06g050550(541); Solyc09g031600(473);  Solyc09g010130(295) |
| Cargo recognition | VPS26 | YJL053W(379) | At4g27690(303); At5g53530(302) | Solyc04g050240(301) |
|  | VPS29 | YHR012W(282) | At3g47810(190) | Solyc03g113020(189) |
|  | VPS35 | YJL154C(944) | At1g75850(790); At2g17790(787);  At3g51310(790) | Solyc02g083560(791); Solyc03g033400(791);  Solyc12g089340(792) |
| ESCRT-I | VPS23 | YCL008C(385) | *At2g38830(331)*; At3g12400(398);  *At5g13860(368)* | Solyc09g009270(404); Solyc09g065770(350);  Solyc10g085590(403) |
|  | VPS28 | YPL065W(242) | *At4g05000(210)*; At4g21560(209); | Solyc02g080270(274); Solyc03g031750(209) |
|  | VPS37 | NF | *At2g36680(218)*; *At3g53120(217)* | Solyc10g083780(233); Solyc10g085810(234) |
| ESCRT-II | VPS22 | YPL002C(233) | At4g27040(250) | Solyc10g006320(251) |
|  | VPS25 | YJR102C(202) | *At4g19003(179)* | Solyc08g076130(179) |
|  | VPS36 | NF | *At5g04920(440)* | Solyc01g096910(438) |
| ESCRT-III | VPS2 | YKL002W(232) | At2g06530(225) | Solyc12g009060(179) |
|  | VPS20 & SNF7a | YMR077C(221) | At5g09260(216); *At5g63880(243)* | Solyc12g096650(227) |
|  | VPS24 | YKL041W(224) | *At3g45000 (200)*; *At5g22950(229)* | Solyc06g074840(223); Solyc11g066260(221) |
|  | SNF7b & | YLR025W(240) | At2g19830(213); At4g29160(219) | Solyc05g026050(218); Solyc08g065900(252);  Solyc11g070120(218) |
|  | SNF7c |  |  |  |
|  | DID2 | YKR035W-A(204) | At1g73030(203); At1g17730(203) | Solyc03g121360(203); Solyc06g062510(203) |
| Misc. | VPS31/  Bro1 | YOR275C(661) | At1g15130(846) | Solyc04g007560(184); Solyc05g008430(184) |
|  | VPS4 | YPR173C(437) | At2g27600(435) | Solyc11g067230(432); Solyc11g067240(432);  Solyc11g007170(404) |
|  | Hrs/  VPS27 | NF | *At1g61690(1171)* | Solyc02g079850(979) |
| Given are the names of the complex, the name used for the factor in yeast, the gene accession number and in brackets the amino acid length of the (co-)orthologues in yeast, *A. thaliana* and *S. lycopersicum*. Underlined accession Ids were used as bait to identify orthologues, accession Ids in italics are bioinformatically identified as per previous studies  NF: not found | | | | |
