# Supplementary material for: The Complexity of Vesicle Transport Factors in Plants Examined by Orthology Search
Source: PLoS One. 2014 May 20;9(5):e97745. doi: 10.1371/journal.pone.0097745 (PMC4028247; doi:10.1371/journal.pone.0097745)
Supplement: Table S17 — The Rab GTPase components of yeast, A. thaliana and tomato identified via OrthoMCL and PGAP. (DOCX) [file pone.0097745.s019.docx]

| **Table S19.** The Rab GTPase components of *A. thaliana* and tomato | | | | | |
| --- | --- | --- | --- | --- | --- |
| **Co.** | **Factors** | **Yeast** | ***A. thaliana*** | ***S. lycopersicum*** | |
| Rab group | RABA1a-e  RABA1g-i  RABA2a-b | YER031C(223)  YGL210W(222) | At1g06400(216-1a); *At1g07410(214-2b)*;  *At1g09630(217-2a)*; At1g16920(216-1b); *At1g28550(218-1i)*; *At2g33870(218-1h)*;  *At3g15060(217-1g)*; *At4g18430(217-1e)*; *At4g18800(214-1d)*; *At5g45750(216-1c)* | Solyc01g103380(214); Solyc02g072180(217); Solyc03g006270(216); Solyc03g079900(215);  Solyc05g052900(215); Solyc06g076450(215); Solyc07g055290(218); Solyc10g008840(215);  Solyc10g045550(215); Solyc12g010790(217);  Solyc12g014080(217) | |
|  | RABA1f | NF | *At5g60860(217-f)* | Solyc07g053480(217); Solyc12g010790(217) | |
|  | RABA2c-d | NF | *At3g46830(217-c)*; *At5g59150(217-d)* | Solyc06g005810(216) | |
|  | RABA3 | NF | *At1g01200(237)* | Solyc01g096220(243) | |
|  | RABA4a | NF | *At5g65270(226-a)* | Solyc04g012180(222); Solyc11g010100(224) | |
|  | RABA4b | NF | *At4g39990(224-b)* | NF | |
|  | RABA4c-d | NF | At3g12160(222-d); *At5g47960(223-c)* | Solyc01g086850(225); Solyc03g064020(224)  Solyc10g079030(226) | |
|  | RABA4e | NF | *At2g22390(176-e)* | NF | |
|  | RABA5a | NF | *At5g47520(221-a)* | Solyc05g054150(226); Solyc11g012460(276) | |
|  | RABA5b | NF | *At3g07410(217-b)* | Solyc09g097900(216) | |
|  | RABA5c-e | NF | *At1g05810(261-e)*;  *At2g31680(219-d)*; At2g43130(214-c) | Solyc09g098170(217) | |
|  | RABA6a-b | NF | *At1g18200(229-a)*; *At1g73640(233-b)* | Solyc03g118820(242) | |
|  | RABB1a | NF | *At4g17160(205-a)* | NF | |
|  | RABB1b | NF | At4g17170(211-b) | Solyc10g007700(211); Solyc12g011130(211) | |
|  | RABB1c | NF | *At4g35860(211-c)* | Solyc02g093530(211) | |
|  | RABC1 | NF | *At1g43890(212)* | Solyc04g064510(209) | |
|  | RABC2a | NF | At5g03530(210-a) | Solyc09g008460(216); Solyc10g086310(209) | |
|  | RABC2b | NF | *At3g09910(205-b)* | NF | |
|  | RABD1 | NF | At3g11730(205) | Solyc01g103370(202) | |
|  | RABD2a-c | YFL038C(206) | At1g02130(203-a); At4g17530(202-c); At5g47200(202-b) | Solyc01g090170(203); Solyc05g052070(203); Solyc05g053940(203); Solyc08g078070(203) | |
|  | RABE1a  RABE1c-e | YFL005W(215) | *At3g09900(218-e)*; At3g46060(216-c);  *At3g53610(216-a)*;At5g03520(216-d);  At5g59840(216) | Solyc04g011360(216); Solyc06g005350(144);  Solyc09g010370(216); Solyc10g086350(216);  Solyc11g073050(216) | |
|  | RABE1b | NF | At4g20360(476-b) | Solyc03g112150(477); Solyc06g071790(450) | |
|  | RABF1  RABF2a-b | YKR014C(234)  YOR089C(210)  YNL093W(220) | At3g54840(202-1); At4g19640(200-2b); At5g45130(200-2a) | Solyc02g036450(200); Solyc02g069370(200);  Solyc02g081380(200); Solyc11g008430(200) | |
|  | RABG1 | NF | *At5g39620(204)* | NF | |
|  | RABG2  RABG3a-f | YML001W(208) | At1g22740(203-3b); At1g49300(206-3e);  At1g52280(206-3d); *At2g21880(212-2)*; At3g16100(206-3c); At3g18820(206-3f); *At4g09720(217-3a)* | Solyc01g109520(206); Solyc03g120750(205);  Solyc04g072060(204); Solyc04g051680(219) | |
|  | RABH1a-e | YLR262C(215) | *At2g22290(207-1d)*; At2g44610(208-1b); At4g39890(214-1c); *At5g10260(207-1e)*;  *At5g64990(213-1a)* | Solyc01g088560(208); Solyc03g078570(207); Solyc05g051570(207) | |
| Other small  GTPases | Arf1-l2 | YBR164C(183) | At2g24765(182) | Solyc07g006640(182) | |
|  | ArfRP1-l | YPL051W(198) | *At5g52210(205)* | Solyc10g074630(193) | |
|  | Arl2-like | NF | *At2g18390(185)* | Solyc07g014650(169) | |
|  | Arl5-like | NF | *At3g22950(183)* | Solyc03g005300(183) | |
|  | Arl8-like | NF | *At3g49870(184)*; *At5g37680(184)*; *At5g67560(184)* | Solyc02g064640(184); Solyc02g092330(184);  Solyc03g043960(184); Solyc11g011040(184) | |
|  |  | NF | *At3g49860(176* | NF | |
| 1 | KEULLE | NF | *At1g12080(138)* | NF |  |
|  | SEC1a,b | YDR164C(724) | *At1g02010(673)*; At1g12360(666); *At4g12120(662)* | Solyc01g091300(525); Solyc08g079520(666) | |
| 2 | VPS45 | YGL095C(577) | At1g77140(569) | Solyc04g064820(568) | |
| 3 | VPS33 | YLR396C(691) | *At3g54860(608)* | Solyc05g055600(597) | |
| 4 | SLY1 | YDR189W(666) | *At2g17980(627)* | Solyc01g112150(620) | |
| Given are the names of the complex, the name used for the factor in yeast, the gene accession number and in brackets the amino acid length of the (co-)orthologues in yeast, *A. thaliana* and *S. lycopersicum*. Underlined accession Ids were used as bait to identify orthologues, accession Ids in italics are bioinformatically identified as per previous studies  Additionally, the nomenclature for the isoform in *A. thaliana* is given in the brackets;  ^Φ^pseudogene; NF: not found; 1…PM (SM family), 2…TGN(SM), 3…LE/vac. (SM), 4…ER-Golgi (SM) | | | | | |
