# Supplementary material for: The Complexity of Vesicle Transport Factors in Plants Examined by Orthology Search
Source: PLoS One. 2014 May 20;9(5):e97745. doi: 10.1371/journal.pone.0097745 (PMC4028247; doi:10.1371/journal.pone.0097745)
Supplement: Table S18 — The Tethering factors of yeast, A. thaliana and tomato identified via OrthoMCL and PGAP. (DOCX) [file pone.0097745.s020.docx]

| **Table S22.** The Tethering factors of A. thaliana and tomato | | | | | | | |
| --- | --- | --- | --- | --- | --- | --- | --- |
| **Com.** | **Factors** | **Yeast** | ***A.thaliana*** | | ***S. lycopersicum*** | | |
| Coiled coils | Uso1 | YDL058W(1790) | At3g27530(914) | Solyc08g081410(839) | | | |
|  | COY1 | YKL179C(679) | At3g18480(689) | Solyc06g069210(684) | | | |
|  | Rud3/Grp1 (YOR216C; 484), Imh1 (YLR309C; 911) | | | NF | | | |
| HOPS (C) | Vps11 | YMR231W(1029) | At2g05170(932) | Solyc06g065170(954) | | | |
|  | Vps16 | YPL045W(798) | At2g38020(858) | Solyc07g056220(843) | | | |
|  | Vps18 | YLR148W(918) | *At1g12470(988)* | Solyc06g005080(1067) | | | |
|  | Vps33 | YLR396C(691) | At3g54860(608) | Solyc05g055600(597) | | | |
|  | Vps39 | YDL077C(1049) | *At4g36630(1000)* | Solyc02g085220(1001); Solyc07g056660(1004) | | | |
|  | Vps41 | YDR080W(992) | *At1g08190(980)* | Solyc06g074740(960); Solyc11g066560(957) | | | |
| 1 | Vps8 | YAL002W(1274) | At4g00800(1913) | Solyc05g044610(1864) | |  | |
|  | Vps3 (YDR495C; 1011) | | | NF | | | |
| CATR family Exocyst (C) | Sec3 | YER008C(1336) | NF | NF | | | |
|  |  | NF | At1g47550(888); *At1g47560(887)* | Solyc12g088990(889) | | | |
|  | Sec5 | YDR166C(971) | At1g21170(1090); At1g76850(1090) | Solyc04g071350(1106); Solyc12g056520(977) | | | |
|  | Sec6 | YIL068C(805) | At1g71820(774) | Solyc04g072290(749) | | | |
|  | Sec8 | YPR055W (1065) | NF | NF | | | |
|  |  | NF | At3g10380(1053) | Solyc11g008340(1144) | | | |
|  | Sec10 | YLR166C(1065) | At5g12370(825) | Solyc03g095410(830); Solyc10g019110(603);  Solyc10g019140(505); Solyc11g050710(770) | | | |
|  | Sec15 | YGL233W(910) | At3g56640(790); At4g02350(787) | Solyc09g075280(804); Solyc12g096290(765) | | | |
|  | Exo70 | YJL085W(623) | *At1g07000(599)*; *At1g07725(615)*; *At1g72470(633)*; *At2g28640(605)*; *At2g28650(573)*; *At2g39380(637)*; *At3g09530(637)*; *At3g55150(636)*; At5g03540(664); *At5g13150(653)*; *At5g52340(631)*; *At5g52350(586)*; *At5g59730(634)*; *At5g61010(639)* | | Solyc01g009880(664); Solyc03g115070(635);  Solyc04g009740(651); Solyc04g077760(722);  Solyc05g024340(592); Solyc05g054820(645);  Solyc06g005280(598); Solyc06g075610(623);  Solyc10g081940(640); Solyc11g006620(658);  Solyc11g073010(634); Solyc12g055770(656) | | |
|  | Exo84 | YBR102C(753) | NF | | NF | | |
|  |  | NF | At5g49830(814) | | Solyc03g123870(773) | | |
| 2 | Tip20 | YGL145W(701) | NF | | NF | |  |
|  |  | NF | At1g08400(804) | | NF | |  |
|  | Dsl1 (YNL258C; 754), Sec39 (YLR440C; 709) | | | | NF | |  |
| CATR family COG complex (C) | COG1 | YGL223C(417) | NF | | NF | | |
|  |  | NF | *At5g16300(1068)* | | Solyc03g120700(1073) | | |
|  | COG2 | YGR120C(262) | NF | | NF | | |
|  |  | NF | *At4g24840(756)* | | Solyc08g006660(742) | | |
|  | COG3 | YER157W(801) | *At1g73430(784)* | | Solyc04g008640(779) | | |
|  | COG4 | YPR105C(861) | *At4g01400(1110)* | | Solyc07g056010(736) | | |
|  | COG5 | YNL051W(403) | NF | | NF | | |
|  |  | NF | *At1g67930(832)* | | Solyc10g007720(845) | | |
|  | COG6 | YNL041C(839) | *At1g31780(680)* | | Solyc08g075440(691) | | |
|  | COG7 | YGL005C(279) | NF | | NF | | |
|  |  | NF | *At5g51430(836)* | | Solyc05g006950(836) | | |
|  | COG8 | YML071C(607) | NF | | NF | | |
|  |  | NF | *At5g11980(569)* | | Solyc01g087650(577) | | |
| 3 | VPS52 | YDR484W(641) | At1g71270(707); At1g71300(721) | | Solyc02g094090(696) | |  |
|  | VPS53 | YJL029C(822) | At1g50500(847); At1g50970(569) | | Solyc04g049490(824) | | |
|  | VPS54 | YDR027C(889) | At4g19490(1034) | | Solyc02g030100(266); Solyc02g069660(292) | | |
|  | VPS51 (YKR020W; 164) | | | | NF | | |
| TRAPP-I | Bet3 | YKR068C(193) | *At5g54750(199)* | | Solyc01g096530(186); Solyc10g006490(186) | | |
|  | Bet5 | YML077W(159) | *At1g51160(169)* | | Solyc03g007680(175) | | |
|  | Trs20 | YBR254C(175) | *At1g80500(135)* | | Solyc06g068030(137) | | |
|  | Trs23 | YDR246W(219) | *At5g02280(141)* | | Solyc01g100840(141) | | |
|  | Trs31 | YDR472W(283) | *At5g58030(195)* | | Solyc04g015050(194) | | |
|  | Trs33 | YOR115C(268) | *At3g05000(173)* | | Solyc04g072410(175) | | |
|  | Trs85 (YDR108W; 698) | | | | NF | |  |
| 4 | Trs120 | YDR407C( 1289) | NF | | NF | |  |
|  |  | NF | At5g11040(1186) | | Solyc06g065840(1186) | |  |
|  | Trs130 | YMR218C(1102) | NF | | NF | |  |
|  |  | NF | *At5g54440(1259)* | | Solyc07g063440(1255) | |  |
|  | Trs65 (YGR166W; 560) | | | | NF | | |
| Given are the names of the complex, the name used for the factor in yeast, the gene accession number and in brackets the amino acid length of the (co-)orthologues in yeast, *A. thaliana* and *S. lycopersicum*. Underlined accession Ids were used as bait to identify orthologues, accession Ids in italics are bioinformatically identified as per previous studies  NF: not found; d.a: Discussed above; 1..CORVET (C), 2…CATR family DSL1 complex (C), 3… CATR family GARP complex (C), 4…Additional TRAPP-II factors | | | | | | | |
