# Supplementary material for: The Complexity of Vesicle Transport Factors in Plants Examined by Orthology Search
Source: PLoS One. 2014 May 20;9(5):e97745. doi: 10.1371/journal.pone.0097745 (PMC4028247; doi:10.1371/journal.pone.0097745)
Supplement: Table S19 — The Q and R-SNARE components of yeast, A. thaliana and tomato identified via OrthoMCL and PGAP. (DOCX) [file pone.0097745.s021.docx]

| **Table S19.** The Q and R-SNARE components of *A. thaliana* and tomato | | | | | | |
| --- | --- | --- | --- | --- | --- | --- |
| **Com.** | **Factors** | **Yeast** | ***A. thaliana*** | | ***S. lycopersicum*** | |
| ER(Qa) | SYP81 | NF | *At1g51740(310)* | Solyc03g033850(280); Solyc04g071730(317) | | |
| 1 | SYP31^a^  SYP32^b^ | YLR026C(340) | At3g24350(361)^b^; At5g05760(336)^a^ | Solyc07g054140(318); Solyc08g067910(341) | | |
| TGN  (Qa) | SYP41^c^  SYP42^d^  SYP43^e^ | YOL018C(397) | At3g05710(331)^e^; At4g02195(323)^d^; At5g26980(322)^c^ | Solyc01g100170(324); Solyc09g075530(319) | | |
| 2 | SYP21^f^  SYP22^g^  SYP23^h^ | YOR036W(288) | At4g17730(262)^h^; At5g16830(279)^f^; At5g46860(268)^g^ | Solyc06g062360(273); Solyc06g072760(283);  Solyc08g005200(273); Solyc08g076540(273);  Solyc11g066910(273) | | |
|  | SYP24 | NF | At1g32270(416) | NF | | |
| cell  plate  (Qa) | KNOLLE^i^ | YMR183C(295)YPL232W(290) | At1g08560(310)^i^; At1g11250(298); At1g61290(303); *At3g03800(306)*; *At3g52400(341)*; At4g03330(305); At5g08080(315) | Solyc01g056810(253); Solyc01g109750(280);  Solyc06g053760(311); Solyc07g052470(305);  Solyc10g081580(310); Solyc12g005580(310) | | |
|  | SYP112 | NF | *At2g18260(305)* | Solyc02g085090(299) | | |
| 3 | PEN1 | NF | At3g11820(346) | Solyc01g006950(300); Solyc10g081850(303) | | |
|  | SYP122 | Same orthology group as KNOLLE | | | | |
| 4 | SYP123 | Same orthology group as KNOLLE | | | | |
|  | SYP124  SYP125 | Same orthology group as KNOLLE | |  | | |
| 5 | SYP131  SYP132 | Same orthology group as KNOLLE | | | | |
| 6 | SEC20 | NF | At3g24315(293); At4g13480(261) | Solyc09g090860(293) | | |
| 7 | MEMB11  MEMB12 | NF | At2g36900(225); At5g50440(219) | Solyc07g047720(245) | | |
| 8 | GOS11 | NF | At1g15880(223) | Solyc06g065740(221) | | |
|  | GOS12 | YHL031C(223) | At2g45200(257) | Solyc01g091020(235) | | |
| TGN/Vac.  (Qb) | VTI11^j^ | NF | At2g36900(225); At5g39510(221)^j^ | Solyc07g047720(245) | | |
|  | VTI12^k^ | NF | *At1g26680(920)^k^*; *At2g24645(490)*; *At2g24650(1045)*; *At2g24680(471)*; *At2g24681(286)*;  *At2g24690(777)*; *At2g24696(375)*; *At2g24700(555)*; *At4g00260(528)*; At4g31610(517); *At4g31615(487)*; *At4g31620(492)*; *At4g31630(512)*; *At4g31640(352)*; *At4g31650(493)*; *At4g31660(251)*; *At4g31680(462)*; *At4g31690(461)*;  *At5g32460(530)*; | Solyc08g006190(544); Solyc08g006200(566);  Solyc08g006240(504); Solyc08g006280(565) | | |
|  | VTI13^l^  VTI14^m^ | YMR197C(217) | At3g29100(195)^l^; At5g39510(221); At5g39630(207)^m^ | Solyc03g115760(221) | | |
| TGN/PM  (Qb) | NPSN12^o^  NPSN13^p^ | NF | At1g48240(265)^o^; *At3g17440(269)^p^* | Solyc12g098950(332) | | |
|  | NPSN11^n^ | NF | At2g35190(265)^n^ | Solyc08g077550(261) | | |
| 9 | USE11^q^  USE12^r^ | NF | *At1g54110(236)^q^*; *At3g55600(240^r^* | Solyc02g069380(239) | | |
| 10 | BET11s BET12t | NF | At3g58170(122)^s^; *At4g14455(130)^t^* | Solyc06g008520(117); Solyc09g089630(145) | | |
| 11 | SFT11^u^  SFT12^v^ | YKL006C-A(97) | *At1g29060(134)^v^*; At4g14600(137)^u^ | Solyc04g074940(133) | | |
| 12 | SYP51^w^  SYP52^x^ | YGL212W(316) | At1g16240(232)^w^; *At1g79590 (233)^x^* | Solyc07g065030(209); Solyc10g081060(223) | | |
| 13 | SYP61 | YDR468C(224) | At1g28490(245) | Solyc10g081060(223); Solyc12g089150(129); Solyc12g014550(153) | | |
| PM  (Qc) | SYP73^I^ | NF | At3g61450(264)^I^ | NF | | |
|  | SYP71^y^  SYP72^z^ | NF | *At3g09470 (464) ^y^; At3g45280(267)^z^* | Solyc09g010470(433) | | |
| PM  (Qbc) | SNAP33 | NF | *At5g61210(300)* | Solyc03g115470(343); Solyc06g069570(306) | | |
|  | SNAP29 | NF | *At5g07880(251)* | NF | |  |
|  | SNAP30 | NF | *At1g13890(263)* | NF | |  |
| Given are the names of the complex, the name used for the factor in yeast, the gene accession number and in brackets the amino acid length of the (co-)orthologues in yeast, *A. thaliana* and *S. lycopersicum*. Underlined accession Ids were used as bait to identify orthologues, accession Ids in italics are bioinformatically identified from previous studies  1…Golgi(Qa); 2…Late Endosome/vac. (Qa); 3…PM1(Qa); 4…PM2(Qa); 5…PM3(Qa); 6…ER(Qb); 7…ER/Golgi(Qb); 8…Golgi(Qb); 9…ER(Qc); 10…ER/Golgi(Qc); 11…Golgi(Qc); 12…TGN/Vac.(Qc); 13…TGN/Endo (Qc); ^Φ^Obsolete TAIR Id; NF: not found; Arabidopsis Ids referred specifically as respective factors have been mentioned in the tables (Bassham et al, 2008) | | | | | | |

| The R-SNARE components of *A. thaliana* and tomato | | | | | |
| --- | --- | --- | --- | --- | --- |
| **Com.** | **Factors** | **Yeast** | ***A. thaliana*** | | ***S. lycopersicum*** |
| 1 | SEC22 | YLR268W(214) | At1g11890(218) | Solyc03g122090(218); Solyc06g063030(218);  Solyc12g049420(218) | |
| 2 | YKT61  YKT62 | YKL196C(200) | At5g58060(221); At5g58180(199); | Solyc07g005200(199) | |
| TGN/Vac^*^  (R) | VAMP711^a^  VAMP712^b^  VAMP713^c^  VAMP714^d^  VAMP723^e^  VAMP728^f^ | YOR327C(115)  YAL030W(117) | *At2g25340(219)^b^*; At2g33110(217)^e^; At3g24890(124)^f^; At4g32150(219)^a^; At5g11150(221)^c^; At5g22360(221)^d^ | Solyc02g069150(222); Solyc06g065950(220)^,^;  Solyc06g074250(261); Solyc09g091610(221);  Solyc12g098180(221) | |
| TGN/PM1^*^  (R) | VAMP722^h^  VAMP725^i^  VAMP726^j^ | NF | At1g04750(219); At1g04760(220)^j^;  *At2g32670(285)^i^*; At2g33120(229)^h^ | Solyc02g069150(222); Solyc06g083530(220); Solyc09g061620(220) | |
|  | VAMP721^g^ | NF | At1g04740(219)^g,Φ^ | NF | |
| 3 | VAMP724 | NF | *At4g15780(222)* | Solyc01g066940(221) | |
| 4 | VAMP727 | NF | At3g54300(240) | Solyc11g006780(240) | |
| PM  (R) | TYN11^k^  TYN12^l^ | YBL106C(1010)  YPR032W(1033) | *At4g35560(1050)^l,^*; *At5g05570(1124)^k^* | Solyc02g082690(1054); Solyc06g051380(355); Solyc06g051390(749); Solyc09g005600(1103) | |
| 5 | Sec18 | YBR080C(758) | At4g04910(742) | Solyc01g104920(692) | |
|  | Sec17 | YBL050W(292) | *At3g56190(289)*; *At3g56450(381)* | Solyc05g052310(243); Solyc06g050770(289) | |
| Given are the names of the complex, the name used for the factor in yeast, the gene accession number and in brackets the amino acid length of the (co-)orthologues in yeast, *A. thaliana* and *S. lycopersicum*. Underlined accession Ids were used as bait to identify orthologues, accession Ids in italics are bioinformatically identified from previous studies, aa lenghts in bold indicate protein sequences with significantly different length compared to the bait  1… ER/Gol.(R), 2…Golgi/Vac(R), 3… TGN/PM2 (R), 4… TGN/PM3 (R), 5… Disassembly unit; *Overlapping orthologues of these factors; ^Φ^Obsolete TAIR Id; NF: not found; Arabidopsis Ids referred specifically as respective factors have been mentioned in the tables (Bassham et al, 2008) | | | | | |
